# Regulation of Chloroplast Protein Import by the Ubiquitin E3 Ligase SP1 Is Important for Stress Tolerance in Plants

**DOI:** 10.1016/j.cub.2015.08.015

**Published:** 2015-10-05

**Authors:** Qihua Ling, Paul Jarvis

**Affiliations:** 1 Department of Plant Sciences, University of Oxford, South Parks Road, Oxford OX1 3RB, UK

## Abstract

Chloroplasts are the organelles responsible for photosynthesis in plants [1, 2]. The chloroplast proteome comprises ∼3,000 different proteins, including components of the photosynthetic apparatus, which are highly abundant. Most chloroplast proteins are nucleus-encoded and imported following synthesis in the cytosol. Such import is mediated by multiprotein complexes in the envelope membranes that surround each organelle [3, 4]. The translocon at the outer envelope membrane of chloroplasts (TOC) mediates client protein recognition and early stages of import. The TOC apparatus is regulated by the ubiquitin-proteasome system (UPS) in a process controlled by the envelope-localized ubiquitin E3 ligase SUPPRESSOR OF PPI1 LOCUS1 (SP1) [5, 6]. Previous work showed that SP1-mediated regulation of chloroplast protein import contributes to the organellar proteome changes that occur during plant development (e.g., during de-etiolation). Here, we reveal a critical role for SP1 in plant responses to abiotic stress, which is a major and increasing cause of agricultural yield losses globally [7]. *Arabidopsis* plants lacking SP1 are hypersensitive to salt, osmotic, and oxidative stresses, whereas plants overexpressing SP1 are considerably more stress tolerant than wild-type. We present evidence that SP1 acts to deplete the TOC apparatus under stress conditions to limit the import of photosynthetic apparatus components, which may attenuate photosynthetic activity and reduce the potential for reactive oxygen species production and photo-oxidative damage. Our results indicate that chloroplast protein import is responsive to environmental cues, enabling dynamic regulation of the organellar proteome, and suggest new approaches for improving stress tolerance in crops.

## Highlights

• Levels of the chloroplast E3 ligase SP1 influence plant abiotic stress tolerance
• Effects of SP1 on stress tolerance are linked to reactive oxygen species levels
• SP1 acts to deplete the chloroplast protein import (TOC) machinery under stress
• TOC depletion by SP1 is linked to reduced plastid import of photosynthesis proteins

## Graphical Abstract

**Figure:**
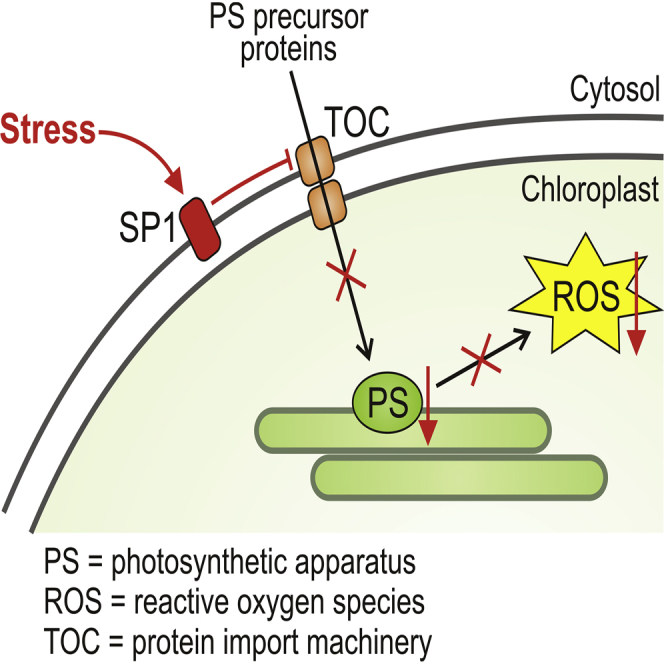

## Teaser

A harmful effect of abiotic stress in plants is photo-oxidation linked to overproduction of reactive oxygen species (ROS) by the photosynthetic machinery. Ling and Jarvis show that the E3 ligase SP1 promotes stress tolerance by depleting the chloroplast protein import apparatus, which limits photosystem assembly and the potential for ROS formation.

## Results and Discussion

### SP1 Expression Levels Influence Abiotic Stress Tolerance

**Figure 1:**
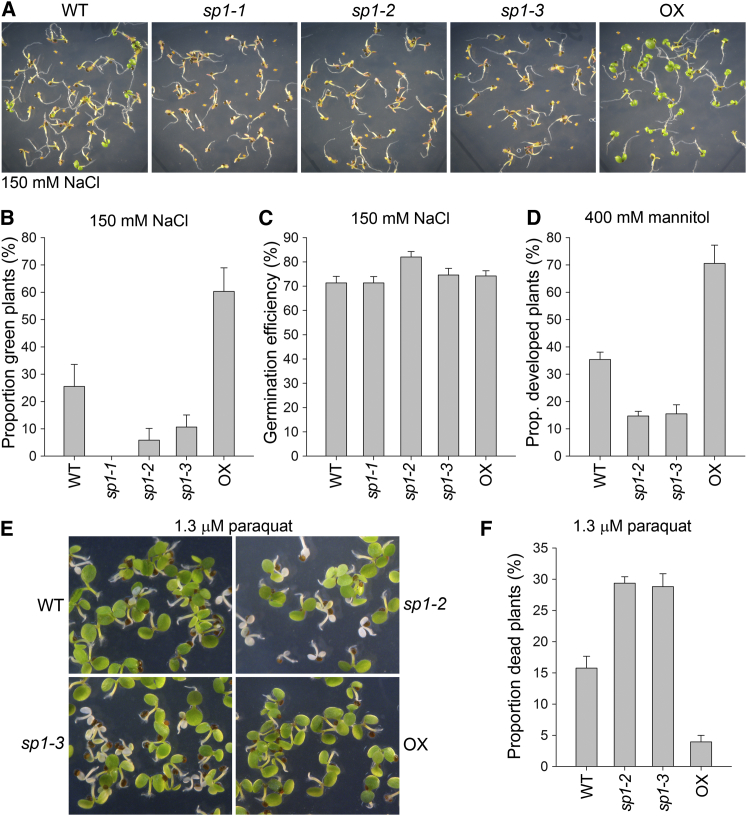
SP1 Expression Levels Influence Abiotic Stress Tolerance (A–C) Plants grown under high-salinity stress were photographed (A) and scored for a measure of stress tolerance (B) and germination efficiency (C). Error bars indicate SEM (n = 3). (D) Plants grown under osmotic stress were scored for a measure of stress tolerance. Error bars indicate SEM (n = 6). (E and F) Plants grown under oxidative stress were photographed (E) and scored for death/survival as a measure of stress sensitivity (F). Error bars indicate SEM (n = 3).

Because SUPPRESSOR OF PPI1 LOCUS1 (SP1) is an important mediator of the chloroplast protein import and proteome changes that occur during plant development [5], we wished to investigate whether this E3 ligase is similarly involved in those changes that occur during plant responses to abiotic stress. This possibility was suggested by reports showing that changes in photosynthetic activity and the chloroplast proteome form an important component of plant stress responses [8, 9]. Thus, we grew *sp1* mutant and SP1 overexpressor (OX) *Arabidopsis* plants under different abiotic stress conditions, starting with high salinity (150 mM NaCl). Mutant plants failed to develop under these conditions, whereas SP1 overexpressors were more stress tolerant than wild-type (Figures 1A and 1B); in neither case could the developmental and greening differences be accounted for by differences in germination efficiency (Figure 1C). Similar results were obtained in relation to osmotic stress (300–400 mM mannitol), using both primary leaf emergence and chlorophyll accumulation as measures of stress tolerance (Figures 1D and S1A–S1C). By contrast, the different genotypes were indistinguishable when grown on normal medium under the same growth conditions (Figure S1D).

An important component of salinity and osmotic stresses is oxidative stress, linked to the overproduction of reactive oxygen species (ROS), which can damage cellular components. Because SP1 resides in chloroplasts, an important source of ROS [8, 9], we suspected that SP1’s role in stress is linked to oxidative effects. To assess this possibility, we subjected the same plant genotypes to oxidative stress induced by the herbicide paraquat (1.3 μM), which interferes with photosynthetic electron transport. As expected, *sp1* mutants showed a higher death rate under these conditions, whereas SP1 overexpressors were more stress tolerant than wild-type (Figures 1E, 1F, and S1E).

Chloroplast protein import is impaired under temperature stress [10]. Thus, we tested the effect of temperature stress on *sp1* mutant and SP1 overexpressor plants, using the reported conditions. However, no obvious differences between the genotypes could be seen (data not shown). We also applied high-light stress using an established method [11], but again, no clear differences between the genotypes were found (data not shown). These results suggest (1) that SP1 is involved in the tolerance of some, but not all, abiotic stresses, (2) that redundant pathways may compensate for the loss of SP1 under certain conditions, or (3) that some stresses, e.g., extreme light, might have consequences so severe or direct that they overwhelm the SP1-dependent mechanism, rendering it unable to cope. The latter two possibilities seem more likely, as *SP1* transcript levels are elevated under various abiotic stresses including not only drought and osmotic stresses but also temperature and light intensity stresses [12]. SP1 may also act in biotic stress, although the mechanisms involved are unknown [13].

### Effects of SP1 on Stress Tolerance Are Linked to ROS Regulation

**Figure 2:**
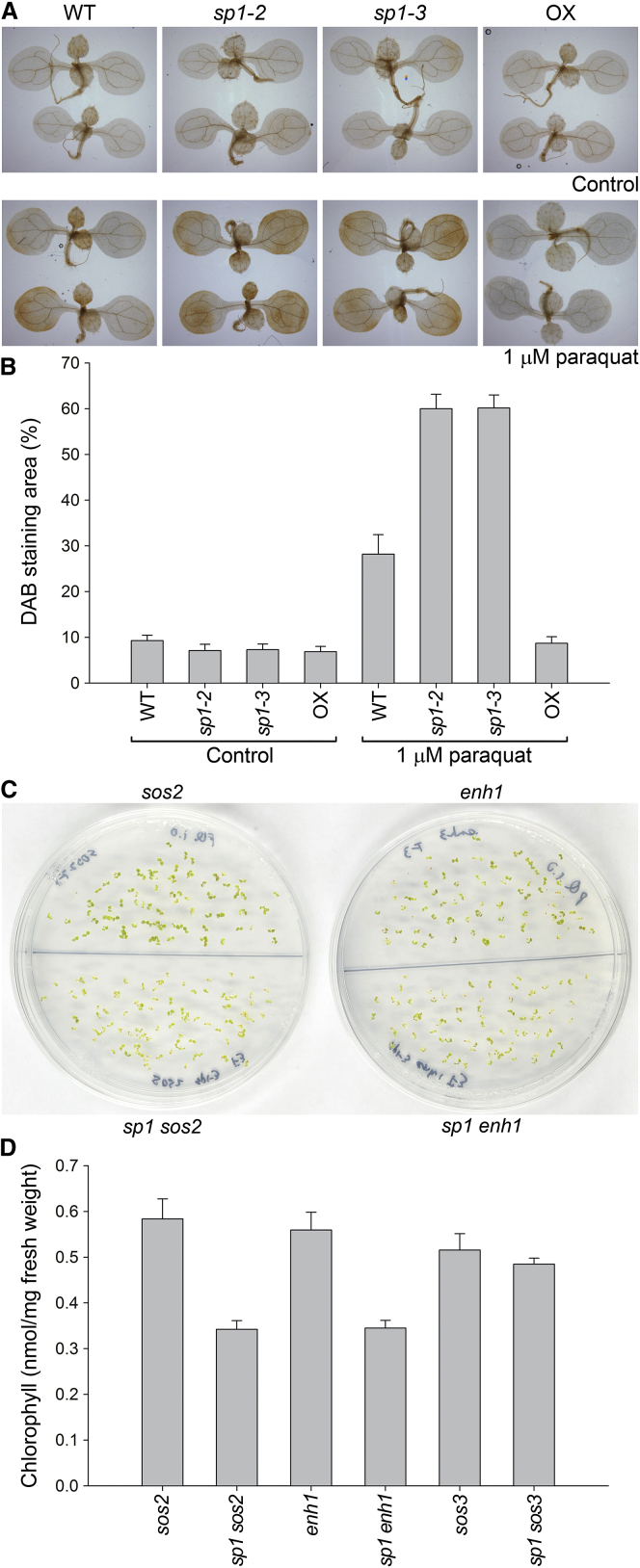
Effects of SP1 on Stress Tolerance Are Linked to ROS (A and B) Staining for hydrogen peroxide accumulation following oxidative stress. Stressed and control, mock-treated plants were stained with 3,3′-diaminobenzidine (DAB), and representative images are shown (A). The area of intense brown staining (indicative of H_2_O_2_ accumulation) was quantified and expressed as a percentage of the total surface area for each plant (B). Error bars indicate SEM (n = 10). (C and D) Genetic interactions between *sp1* and the salt sensitivity mutations *sos2*, *enh1*, and *sos3*. Plants subjected to moderate oxidative stress were photographed (C) or subjected to chlorophyll content analysis as a measure of phenotype severity (D). Error bars indicate SEM (n = 3).

Accumulation of the purple pigment anthocyanin occurred under salt stress in all genotypes except the SP1 overexpressor (Figure S2A). As anthocyanin acts as an antioxidant and is a sign of ROS overproduction [14], we hypothesized that SP1’s role under stress is to control ROS levels. To directly assess whether the effects of SP1 on stress tolerance are linked to ROS, we stained stressed plants with 3,3′-diaminobenzidine (DAB), which detects hydrogen peroxide, a stable and frequently analyzed ROS molecule [15]. Under all three stress conditions (salinity, osmotic, and oxidative), we observed enhanced DAB staining in *sp1* mutants and reduced staining in SP1 overexpressor plants, relative to wild-type (Figures 2A, 2B, S2B, and S2C). In fact, stressed SP1 overexpressors were indistinguishable from unstressed control plants in relation to DAB staining (Figures 2A and 2B), implying that SP1 overexpression provides a high level of stress tolerance. Thus, the stress tolerance effect of SP1 is inversely correlated with levels of an important ROS.

Previously, *sp1* mutant and SP1 overexpressor plants were shown to exhibit plastid-linked developmental differences under certain challenging conditions (applied for the induction of de-etiolation and leaf senescence) [5]. This raises a question about whether the stress sensitivity and ROS accumulation differences seen here were due to inherent developmental differences between the genotypes. However, mutant and overexpressor plants displayed no obvious growth defects or ROS accumulation differences under the normal (unchallenging) growth conditions employed here (Figures S1D and 2A). Thus, the observed SP1-dependent stress effects were indeed stress-specific responses and unlikely to be the result of intrinsic growth differences between the genotypes.

To further investigate the link between SP1 and oxidative stress, we crossed *sp1* to three well-characterized salt-sensitive mutants, two of which have effects linked to ROS, and identified the corresponding double mutants [16, 17]. The *salt overly sensitive2* (*sos2*) mutation affects a kinase that controls activity of the plasma membrane Na^+^/H^+^ antiporter SOS1 and has an additional role in ROS regulation, whereas *enhancer1 of sos3* (*enh1*) affects a chloroplast protein with possible electron carrier function in ROS detoxification. The *sos3* mutation also affects SOS1 activity, in response to cytosolic calcium, but unlike *sos2*, its role seems to be restricted to ion homeostasis. Genetic analyses imply that the SOS2 and ENH1 proteins work in the same pathway of ROS regulation [17]. In accordance with our preceding results, the *sp1 sos2* and *sp1 enh1* double mutants were even more sensitive to oxidative stress than the already sensitive *sos2* and *enh1* single mutants (Figures 2C and 2D). Enhanced sensitivity of the double mutants cannot be attributed to simple phenotype additivity, as *sp1* single mutants were indistinguishable from wild-type under the moderate stress conditions employed (1 μM paraquat; Figures S2D and S2E). By contrast, *sp1 sos3* double mutants were not significantly more sensitive to oxidative stress than *sos3* (Figure 2D). Synergistic interactions similar to those seen here between *sp1* and either *sos2* or *enh1* are normally observed when two key components of the same pathway are both defective [18], and so we conclude that the role of SP1 in abiotic stress responses is closely connected to ROS regulation and involves a different mechanism from SOS2 or ENH1. There are two general mechanisms whereby plants regulate ROS: scavenging and avoidance [19]. SOS2 and ENH1 are thought to act in ROS scavenging [17], and so SP1 might act in avoidance. Avoidance strategies serve to reduce the production of ROS, for example by repressing photosynthesis.

### Effects of SP1 on Stress Tolerance Are Linked to the Chloroplast Protein Import Machinery

**Figure 3:**
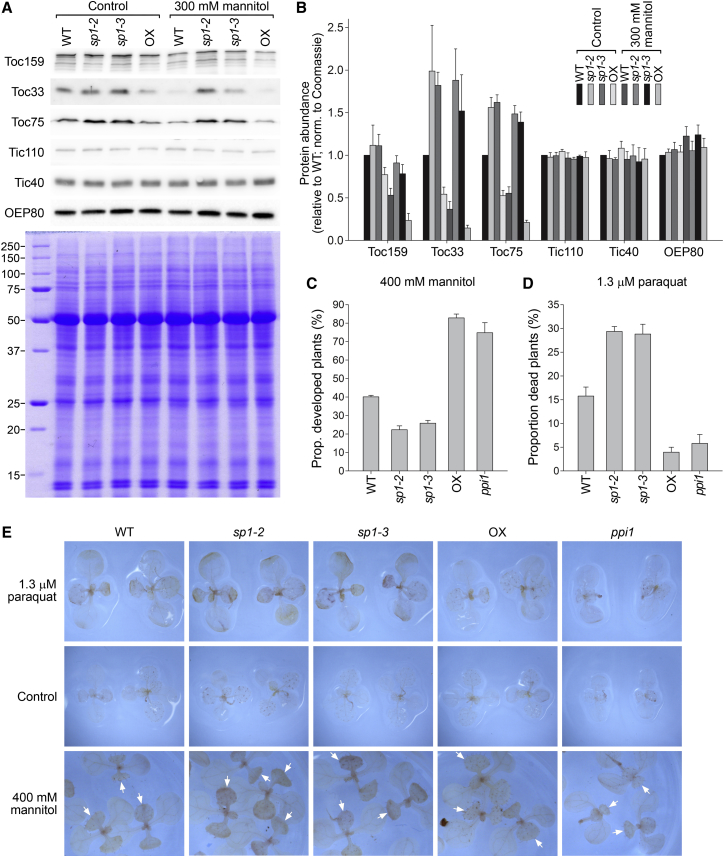
Effects of SP1 on Stress Tolerance Are Linked to the Chloroplast Protein Import Machinery (A and B) Response of TOC protein levels to short-term osmotic stress. Total protein extracts from stressed and control plant material were analyzed by immunoblotting and staining with Coomassie Brilliant Blue (A), and specific bands were quantified (B). Error bars indicate SEM (n = 4). (C–E) Abiotic stress tolerance of the chloroplast protein import mutant *ppi1*. Analyses of osmotic and oxidative stress responses were conducted as in Figure 1. Error bars indicate SEM (n = 3 or 4). Stressed and control, mock-treated plants were stained with DAB, and representative images are shown (E). Following osmotic stress, DAB staining occurred mainly in the true leaves (see arrows).

We wished to understand the molecular basis for the stress tolerance mediated by SP1. Because the primary function of SP1 is to control translocon at the outer envelope membrane of chloroplasts (TOC) protein abundance [5], we began by examining the levels of important components of the chloroplast protein import machinery, in *sp1* mutant and SP1 overexpressor plants, under stress conditions. For this work, we focused on moderate, short-term osmotic stress (established seedlings were transferred to 300 mM mannitol for 2 days), to ensure viability of the plants, to avoid strong morphological changes that might have pleiotropic consequences, and to aid identification of the primary effects of stress. The results revealed that TOC protein levels decline markedly in response to stress in the wild-type (Figures 3A and 3B). This response was SP1 dependent, as it did not occur in *sp1* mutants, whereas the TOC components reached even lower levels in SP1 overexpressor plants. In contrast with the TOC proteins, components of the translocon at the inner envelope membrane of chloroplasts (TIC) did not change in abundance in response to stress, nor did an outer membrane protein uninvolved in the protein import mechanism, outer envelope protein 80 (OEP80) [3, 4, 20] (Figures 3A and 3B). Moreover, inspection of the protein banding pattern following staining with Coomassie revealed that the effect on TOC proteins was unlikely to be a general, damage-related response affecting many proteins (Figure 3A). qRT-PCR analyses did not reveal significant changes in TOC transcript levels under the conditions employed, suggesting a post-translational effect, which is consistent with the aforementioned dependency of the protein changes on SP1 (Figures S3A and S3B).

Under the short-term stress conditions described above, the abundance of photosynthetic apparatus components was unchanged (data not shown). However, following more-prolonged stress treatment, the abundance of such proteins declined markedly and the magnitude of this response was SP1 dependent (Figures S3C and S3D). Thus, the data suggested a hypothesis in which SP1 acts to deplete the TOC apparatus under stress in order to limit the import of components of the photosynthetic machinery. It is well known that photosynthesis-related genes are transcriptionally downregulated under stress [9]. These two aspects of regulation (i.e., transcriptional and post-translational) might have the same eventual consequence of attenuated photosynthesis, reducing the potential for ROS overproduction and photo-oxidative damage, thereby promoting stress tolerance [21, 22]. Suppression of nuclear photosynthetic genes is mediated by retrograde chloroplast-to-nucleus signals [1], and this might only be efficient over the long term. By contrast, SP1-mediated regulation would occur directly at the outer envelope (an ideal position facing both the cytosol and the chloroplast) [5], rapidly initiating responses to changes in the cytosolic and/or chloroplast environments post-translationally during stress. This would explain why TOC proteins are more quickly depleted than other chloroplast components.

The above hypothesis predicts that a TOC mutant with reduced capacity to import photosynthesis-related proteins, such as *plastid protein import1* (*ppi1*) [23], would show abiotic stress tolerance similarly to SP1 overexpressor plants. To investigate this possibility, we grew *ppi1* plants under the osmotic and oxidative stress conditions used previously. In accordance with the hypothesis, *ppi1* plants showed similar levels of stress tolerance to SP1 overexpressors, under both conditions (Figures 3C and 3D). Moreover, in each case, DAB staining revealed ROS levels to be similarly low in *ppi1* mutant and SP1 overexpressor plants (Figure 3E).

Although *ppi1* mutant and SP1 overexpressor plants display similar degrees of stress tolerance, *ppi1* mutants are pale yellow and considerably less vigorous under normal conditions than green SP1 overexpressors [5, 24]. This may be explained as follows: TOC functionality is constitutively impaired in *ppi1* (a single core TOC component, Toc33, is permanently missing), whereas in SP1 overexpressor plants, the effect is more nuanced and more balanced across the different TOC components; SP1 overexpression may increase survival rates with a smaller overall sacrifice of photosynthetic performance. Thus, whereas TOC component knockout mutations such as *ppi1* are unlikely to find stress-related applications in agriculture, SP1 overexpression may be an effective strategy in generating crops better able to cope with abiotic stresses linked to climate change, soil salinification, and other anthropogenic effects [7, 25].

### Elevated SP1 Levels Can Reduce the Import of Photosynthesis-Related Proteins

**Figure 4:**
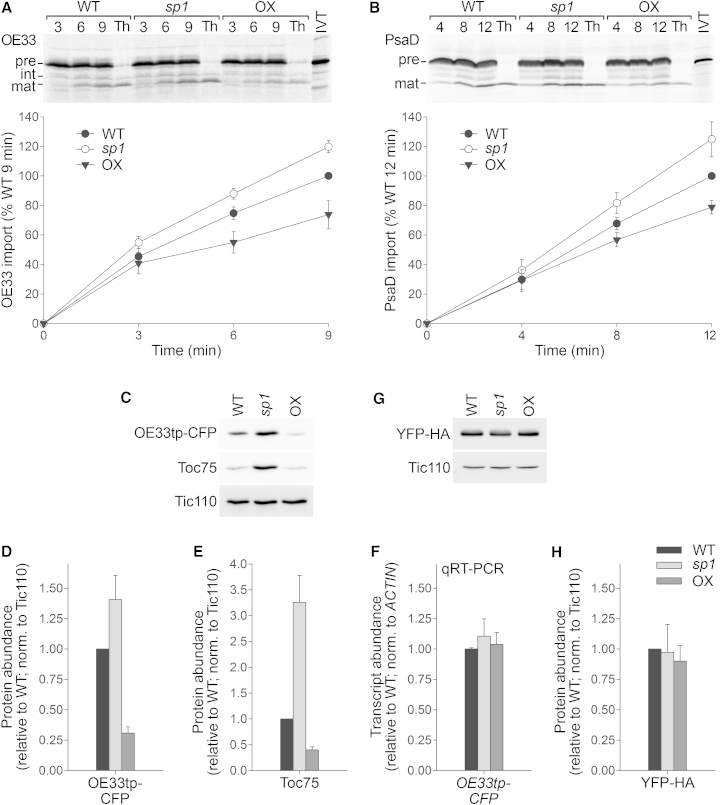
SP1 Influences the Import of Photosynthesis-Related Proteins (A and B) In vitro protein import analysis using chloroplasts isolated from osmotically stressed plants. Import was allowed to proceed for the times shown, and then precursor (p), intermediate (i), and mature (m) protein forms associated with the chloroplasts were analyzed by SDS-PAGE and phosphorimaging. Representative images are shown. IVT, in vitro translation product used in each case; Th, thermolysin protease treatment. Bands corresponding to imported proteins were quantified. Error bars indicate SEM (n = 4 or 5). (C–H) In vivo protein import analysis. Protoplasts were transfected with plasmids encoding either OE33tp-CFP or YFP fused to the hemagglutinin tag (YFP-HA) as a control. Immunoblotting was used to detect the transiently expressed proteins as well as native Toc75 or Tic110 (C and G). The band shown for OE33tp-CFP corresponds to the mature protein generated following transit peptide cleavage, as judged by size comparison with YFP-HA (data not shown). Specific bands were quantified (D, E, and H). In addition, RNA extracted from protoplast samples identical to those shown in (C) were subjected to qRT-PCR analysis using *CFP*- and *ACTIN*-specific primers (F). Error bars indicate SEM (n = 4 or 5 for D, E, and H; n = 3 for F).

Implicit in the aforementioned hypothesis of import regulation as a component of plant stress response is a requirement that SP1 activity should be elevated under stress conditions. The SP1 protein is autoregulated [5], and its abundance is maintained at extremely low levels, so that we are hardly able to detect it by immunoblotting (data not shown). Nonetheless, *SP1* transcript levels are elevated under stress conditions [12], and we therefore assume that protein levels are similarly increased. Activity and/or stability of SP1 might also be regulated dynamically by post-translational modification, which is common for E3 ligases [26]. To test whether altered SP1 levels, and corresponding TOC protein changes, can indeed influence chloroplast protein import efficiency, we compared the import capabilities in vitro of chloroplasts isolated from wild-type, *sp1* mutant, and SP1 overexpressor plants kept under either normal or stress conditions. The chloroplasts were incubated with precursors of the 33-kD subunit of the oxygen evolving complex (OE33) of photosystem II and of subunit D of photosystem I (PsaD), and import was assessed by quantifying the amount of mature, processed protein in the organelles (the identity of which was confirmed by resistance to thermolysin protease treatment [27]) (Figures 4A, 4B, S4A and S4B). The results showed that import of both proteins into SP1 overexpressor chloroplasts was reduced, relative to wild-type, under both conditions. Although *sp1* mutant chloroplasts had import capacity similar to the wild-type in the absence of stress (Figures S4A and S4B), implying that in young seedlings under such conditions the loss of SP1 has minimal consequence, the importance of SP1 was clearly demonstrated following stress treatment as *sp1* mutant chloroplasts displayed elevated import efficiency under these conditions, relative to wild-type (Figures 4A and 4B). These data support the notion that changing SP1 activity acts to regulate the import of photosynthetic machinery components and thus influences stress tolerance. The differing performance of *sp1* mutant chloroplasts under the two conditions probably reflects the fact that TOC abundance differences between wild-type and *sp1* are much more pronounced under stress conditions (Figures 3A and 3B).

To corroborate in a cellular context the results obtained with isolated chloroplasts, we conducted in vivo import experiments based on the transient expression of a chimeric precursor protein (comprising the transit peptide of OE33 fused to CFP; OE33tp-CFP) in transfected protoplasts of each genotype. Chloroplast import of the transiently expressed protein was assessed by monitoring the amount of its mature, processed form by immunoblotting (Figures 4C and 4D). In agreement with the in vitro experiments, chloroplast import was significantly reduced in SP1 overexpressor cells relative to wild-type, providing further support for our hypothesis of SP1-mediated import regulation. In addition, *sp1* protoplasts displayed elevated levels of protein import, which is consistent with the in vitro assays conducted using stressed plants (as protoplastation and transfection are stressful, employing 500 mM mannitol, this is the more-appropriate comparison; see below).

Import capacity differences between the genotypes observed in vivo reflected differences in the abundance of a key TOC component, Toc75, which forms the central, protein-conducting channel (Figures 4C and 4E). This implies that SP1-dependent import regulation affects a broad spectrum of plastid proteins and not just photosynthesis-related proteins, as Toc75 is a general import channel. Thus, SP1-mediated regulation of import under stress conditions may influence other adaptive mechanisms that depend on plastid proteins [28, 29], in addition to photosynthesis. Incongruence of the in vivo and in vitro (normal conditions) import data sets for the *sp1* mutant may be explained in terms of TOC abundance differences, in turn linked to the stress of protoplastation and/or to the age of the leaf material employed (older rosette leaves were used in the transfection studies, whereas young seedlings were employed for chloroplast isolation). The *sp1* mutation has only a moderate effect on TOC levels in young seedlings growing under normal conditions, but as leaves age, TOC proteins gradually accumulate in the absence of SP1 activity [5] (Figures 4C and 4E), and this may be partly responsible for the elevated import capacity seen for *sp1* in the in vivo assay. The stress of protoplastation may also cause TOC protein abundance differences between *sp1* and wild-type in the in vivo assay (paralleling the effect seen in osmotically stressed plants; Figures 3A and 3B), making the in vitro import results for stressed plants a more-valid comparison. Overall, these data further confirm that TOC protein levels, controlled by SP1, are positively correlated with protein import efficiency.

Differences in the abundance of the transiently expressed protein between genotypes were not linked to transfection or expression inconsistencies between the genotypes, as the expression level of *OE33tp-CFP* was similar in the different genotypes, as revealed by qRT-PCR (Figure 4F). In addition, in separate experiments, a control protein similar to the mature form of OE33tp-CFP, but not targeted to chloroplasts (YFP-HA), was expressed equally in all genotypes (Figures 4G and 4H). Analysis of transfected protoplasts by confocal microscopy confirmed that OE33tp-CFP was targeted to chloroplasts in all three genotypes, with efficiencies concordant with those seen by immunoblotting, and that YFP-HA remained in the cytosol (Figures S4C and S4D).

The data indicate that SP1 function is particularly important under challenging conditions when the optimization of import rates becomes critical. Such circumstances may include external stress, as elaborated above, or endogenous defects in chloroplast biogenesis (Figures S4E and S4F).

### Conclusions

Our results provide a mechanistic basis for the regulation of chloroplast protein import in response to environmental stress signals, with the SP1 E3 ligase being a central component of the regulatory mechanism. Furthermore, they clearly demonstrate the physiological significance of such regulation. When the data are considered in conjunction with previous results showing that protein import is regulated in response to temperature and developmental cues [5, 10, 30], a picture of chloroplast protein import as a dynamically regulated process emerges. Such regulation may help to finely tune the composition of the chloroplast proteome, ensuring that it is optimally matched to varying environmental and developmental circumstances. It would complement well-known nuclear photosynthetic gene expression responses, so that both transcriptional and post-translational regulatory steps are required for adaptation to environmental changes. That SP1 overexpression substantially reduces ROS accumulation under stress and significantly promotes stress tolerance (as measured by the greening, developmental progression, and survival) suggests potential applications in agriculture that may help to achieve important food security targets in an increasingly uncertain future [7, 25].

## Supplemental Information

Document S1. Supplemental Experimental Procedures and Figures S1–S4

Document S2. Article plus Supplemental Information
