# Supplementary material for: Regulation of Chloroplast Protein Import by the Ubiquitin E3 Ligase SP1 Is Important for Stress Tolerance in Plants
Source: Curr Biol. 2015 Oct 5;25(19):2527–34. doi: 10.1016/j.cub.2015.08.015 (PMC4598742; doi:10.1016/j.cub.2015.08.015)
Supplement: Document S1. Supplemental Experimental Procedures and Figures S1–S4 [file mmc1.pdf]

Current Biology
Supplemental Information

Regulation of Chloroplast Protein Import by the Ubiquitin E3 Ligase SP1 Is Important for Stress Tolerance in Plants

Qihua Ling and Paul Jarvis

Figure S1

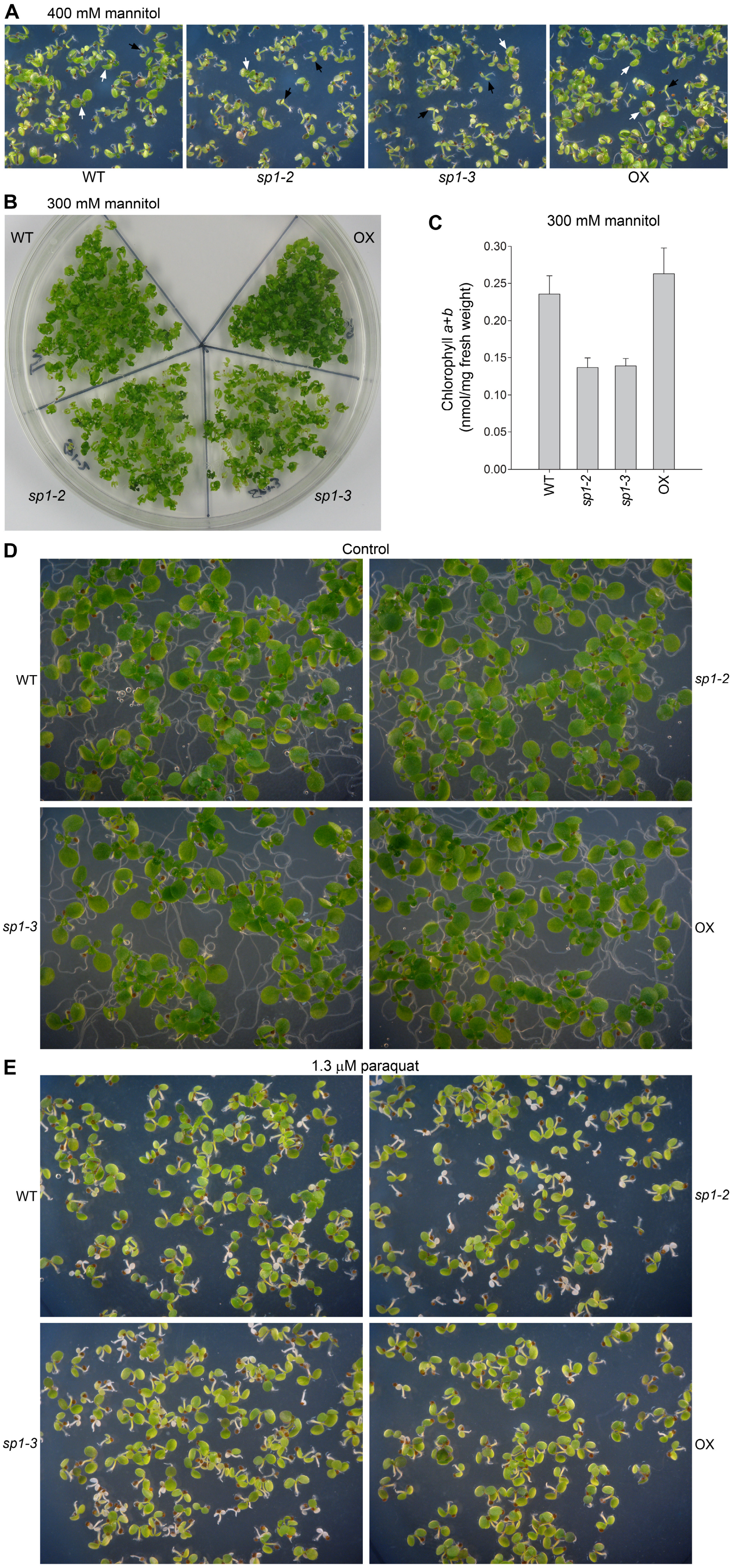

A
400 mM mannitol
WT
sp1-2
sp1-3
OX
B
300 mM mannitol
WT
OX
sp1-2
sp1-3
C
300 mM mannitol
Chlorophyll a+b
(nmol/mg fresh weight)
0.30
0.25
0.20
0.15
0.10
0.05
0.00
WT
sp1-2
sp1-3
OX
D
Control
WT
sp1-2
sp1-3
OX
E
1.3 μM paraquat
WT
sp1-2
sp1-3
OX

Figure S1. SP1 Expression Levels Influence Abiotic Stress Tolerance

(A) Tolerance of strong osmotic stress. Wild type, *sp1* mutant, and SP1 overexpressor (OX) plants were germinated on medium containing 400 mM mannitol, and the plants were allowed to grow for 3 weeks prior to photography (A) and scoring for a measure of stress tolerance (i.e., development to the point of primary leaf emergence; see Figure 1D). Typical developed plants are indicated with white arrows; typical undeveloped plants are indicated with black arrows.

(B and C) Tolerance of moderate osmotic stress. The same genotypes were germinated on medium containing 300 mM mannitol, to apply a more moderate stress, and the plants were allowed to grow for 3 weeks prior to photography (B) and scoring for an alternative measure of stress tolerance (C). In this case, stress tolerance was estimated by quantifying the amount of chlorophyll per unit fresh weight in the plants, which was not possible at higher mannitol concentrations due to the failure of many plants to develop. Values shown are means (n=3), and error bars indicate SEM.

(D and E) Appearance of *sp1* mutant and sp1 overexpressor plants under normal and oxidative stress conditions. The indicated genotypes were germinated on standard MS medium (D) or on medium containing 1.3 µM paraquat (E), and the plants were allowed to grow for 10 days prior to photography. Under the normal (non-stressful) growth conditions applied in D, neither the mutant nor the overexpressor plants displayed any phenotypic differences from wild type. Under the oxidative stress conditions applied in E, a variable proportion of the plants died (dead plants appear small and white) dependent upon the level of SP1 activity. Images shown in Figure 1E correspond to small regions of the images shown in E here, which were selected as being representative of the trends revealed in Figure 1F.

Figure S2

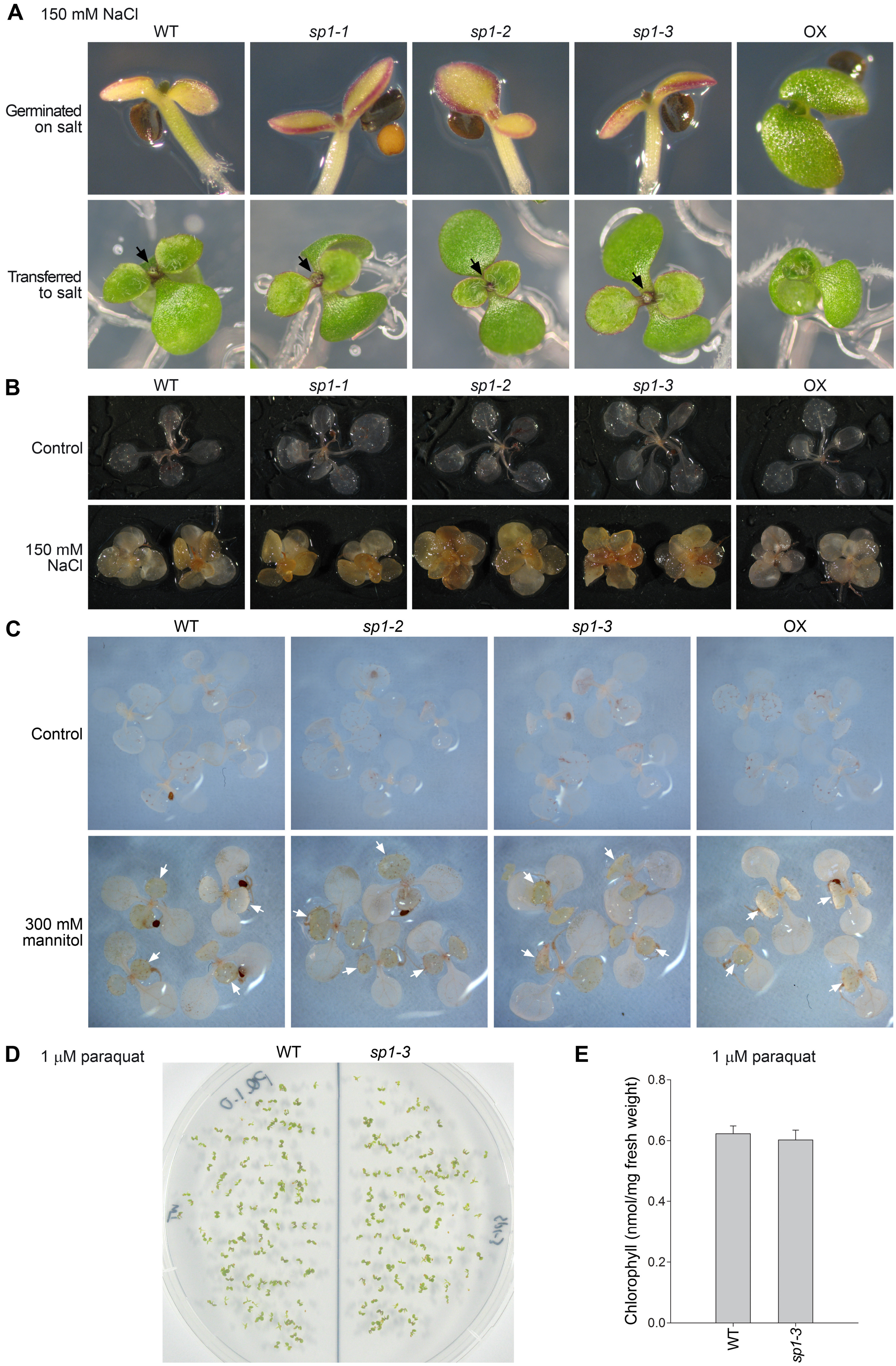
A
150 mM NaCl
WT
sp1-1
sp1-2
sp1-3
OX
Germinated on salt
Transferred to salt
B
WT
sp1-1
sp1-2
sp1-3
OX
Control
150 mM NaCl
C
WT
sp1-2
sp1-3
OX
Control
300 mM mannitol
D
1 μM paraquat
WT
sp1-3
E
1 μM paraquat
Chlorophyll (nmol/mg fresh weight)
0.8
0.6
0.4
0.2
0.0
WT
sp1-3

Figure S2. Effects of SP1 on the Tolerance of Salt and Osmotic Stresses are Linked to ROS

(A) Anthocyanin accumulation following salt stress. The indicated genotypes were germinated on medium containing 150 mM NaCl, or on standard medium and allowed to grow for 4 days before transferral to 150 mM NaCl. Respectively, the plants were allowed to grow for 14 days (upper panels), or for 10 further days following transfer (lower panels). In both cases, accumulation of the purple, stress-related pigment anthocyanin (an antioxidant) was clearly apparent in the leaves of wild type and *sp1* mutant plants, but not in SP1 overexpressor (OX) plants; in the transferred plants, such pigmentation was particularly prominent in the newly emerging true leaves (see arrows). Following germination on saline medium, wild-type and *sp1* mutant plants typically failed to green and were stunted, whereas OX plants were remarkably healthy. Moreover, we consistently observed that SP1 overexpressor plants experienced a developmental delay immediately following transfer to salt stress conditions (hence the smaller size of the OX plant shown). This observation is consistent with our hypothesis that SP1 acts to attenuate photosynthetic activity under stress conditions, which may initially retard growth but ultimately proves beneficial if the stress persists.

(B) Staining for hydrogen peroxide accumulation following salt stress. The same genotypes were stressed exactly as described in A (lower panels), by transferral to medium containing 150 mM NaCl (or to medium lacking NaCl as a control), except that in this case the transferred plants were allowed to grow on for 3 weeks prior to analysis. Stressed and control, mock-treated plants were stained with 3,3'-diaminobenzidine (DAB), and representative images are shown. Brown DAB staining is indicative of H_2O_2 accumulation.

(C) Staining for hydrogen peroxide accumulation following osmotic stress. The indicated genotypes were germinated on standard medium and allowed to grow for 8 days before transferral to medium containing 300 mM mannitol (or medium lacking mannitol as a control). The transferred plants were kept under standard conditions for a further 3 days prior to analysis. Stressed and control, mock-treated plants were stained with DAB, and representative images are shown. Under these conditions, staining was most apparent in the true leaves (see arrows).

(D and E) phenotype of *sp1* single mutant plants under moderate oxidative stress. Wild-type and *sp1* single mutant plants were germinated on medium containing 1 μM paraquat and allowed to grow for 7 days (i.e., identical conditions to those employed in Figures 2C and 2D). At the end of the treatment period, the plants were photographed (D) or subjected to chlorophyll content analysis (E). Values shown in E are means (n=3), and error bars indicate SEM. Phenotypic similarity of the *sp1* mutant to wild type under these conditions indicates that the enhanced ROS sensitivity seen in the *sp1 sos2* and *sp1 enh1* double mutants (Figures 2C and 2D) was synergistic and not an additive effect, and this implies involvement of SP1 in ROS regulation.

Figure S3

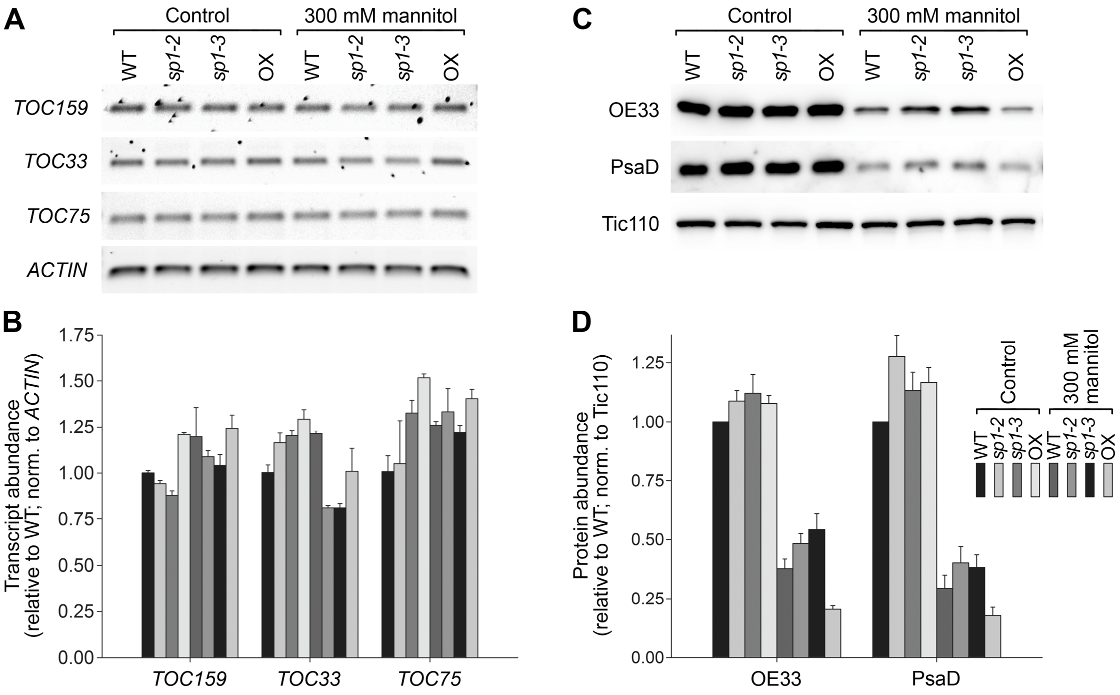

Figure S3. Analyses of the Levels of TOC Transcripts and of Photosynthetic Apparatus Proteins Under Stress Conditions.

(A and B) TOC component mRNA levels do not change in response to osmotic stress. The indicated genotypes were germinated on standard medium and allowed to grow for 7 days before transferral to medium containing 300 mM mannitol (or medium lacking mannitol as a control). The transferred plants were kept under standard conditions for a further 2 days, to deliver short-term osmotic stress as in Figure 3A, prior to analysis. Total RNA was extracted from each of the samples, and subjected to both semi-quantitative (A) and quantitative (B) RT-PCR analysis. Equivalent actin data were employed for normalization purposes. Shading of the bars in B is as defined in panel D.

(C and D). SP1-dependent depletion of photosynthetic proteins under long-term osmotic stress. The indicated genotypes were germinated on standard medium and allowed to grow for 4 days before transferral to medium containing 300 mM mannitol (or medium lacking mannitol as a control). The transferred plants were kept under standard conditions for a further 10 days prior to analysis. This longer-term osmotic stress treatment was designed to enable detection of secondary effects of the stress. Total protein extracts were prepared from the plant material, and equal amounts from each genotype were analysed by immunoblotting using antibodies raised against a core component of each photosystem (PSII: OE33 [PsbO]; PSI: PsaD) (C). Specific bands detected by immunoblotting in C (and in five additional, similar experiments) were quantified, and the values obtained were normalized relative to corresponding data for Tic110; data for each genotype were normalized relative to the wild type (D). Tic110 levels were found to be remarkably stable across the genotypes and conditions, in this study (Figure 3) and in previous studies [S1], and so normalizing relative to Tic110 was justified. Values shown are means (n=6), and error bars indicate SEM.

Figure S4

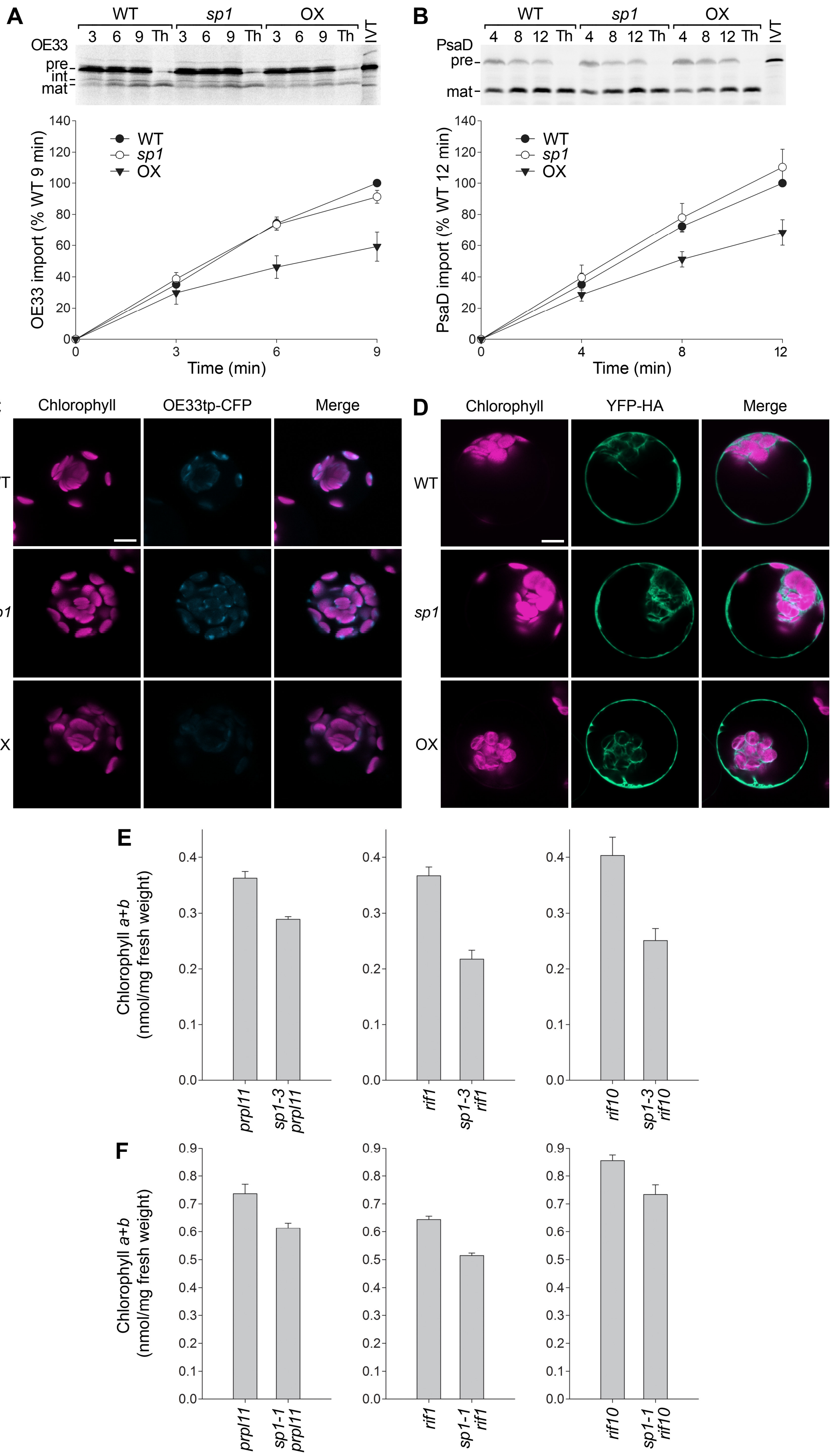

A
WT
sp1
OX
OE33
3 6 9 Th 3 6 9 Th 3 6 9 Th
IVT
pre
int
mat
OE33 import (% WT 9 min)
Time (min)
B
PsaD
4 8 12 Th 4 8 12 Th 4 8 12 Th
PsaD import (% WT 12 min)
C
Chlorophyll
OE33tp-CFP
Merge
D
YFP-HA
E
Chlorophyll a+b (nmol/mg fresh weight)
prpl11
sp1-3 prpl11
rif1
sp1-3 rif1
rif10
sp1-3 rif10
F
sp1-1 prpl11
sp1-1 rif1
sp1-1 rif10

Figure S4. Effects of Altered SP1 Levels on Chloroplast Protein Import and Genetic Interactions between *sp1* and Mutations Affecting the Plastid Genetic System

(A and B) Analysis of protein import in vitro under normal conditions. Chloroplasts isolated from unstressed 10-day-old wild-type, *sp1-3* and SP1 overexpressor (OX) seedlings (normalized according to chlorophyll amount) were incubated with in vitro translated, radiolabelled precursors of OE33 (A) and PsaD (B) under import conditions. Import was allowed to proceed for the time periods shown, and then the precursor (p), intermediate (i) and mature (m) protein forms associated with the chloroplasts were analysed by SDS-PAGE and phosphorimaging. Representative images are shown. The final time-point in each case was conducted in duplicate, with one sample being treated with thermolysin (Th) protease to remove un-imported precursor. The last lane shows an aliquot of the in vitro translation (IVT) product used in each case. Bands corresponding to the intermediate and mature forms of OE33, and to the mature form of PsaD, were quantified, and the values were normalized relative to those obtained for wild-type chloroplasts at the final time-point. Values shown are means (n=3-6), and error bars indicate SEM.

(C and D) Confirmation of the subcellular localization of fluorescent proteins following transfection for the in vivo import analysis. Protoplasts isolated from wild-type, *sp1-3* and SP1 overexpressor (OX) rosette leaves were transfected with plasmids encoding the following chimeric proteins: transit peptide of OE33 fused to CFP (OE33tp-CFP; C), and YFP fused to the haemagglutinin tag (YFP-HA; D). Transfected protoplasts were incubated for ~15 hours prior to analysis by confocal microscopy. Chlorophyll autofluorescence was employed to determine the localization of the CFP and YFP fluorescent signals relative to chloroplasts: CFP was clearly chloroplast localized, whereas YFP was cytosolic. The intensity of CFP signals was dependent upon the protoplast genotype (highest in *sp1* mutant; lowest in SP1 overexpressor, OX), whereas YFP signal intensity was independent of genotype. Scale bars represent 10 µm.

(E and F) Genetic interactions between *sp1* and mutations affecting the plastid genetic system. The *sp1-1* and *sp1-3* mutants were crossed to three different mutants with defects in aspects of plastid gene expression: *prpl11*, *rif1* and *rif10* [S2, S3, S4]. Double homozygous mutants were identified, and their phenotypes were carefully compared with those of the corresponding single mutants after 7 (E) and 10 (F) days of growth, by making chlorophyll measurements. The chlorotic phenotype of each plastid gene expression mutant was significantly enhanced by the *sp1* mutation (which in isolation does not cause any visible abnormal phenotypes under normal growth conditions). It has been shown that the inhibition of plastid gene expression can trigger retrograde signalling to suppress the expression of photosynthetic genes, which is similar to the consequence of stress treatment [S5, S6]. However, this down-regulation is not very efficient when the defect is not strong, as illustrated by the *prpl11* single mutant [S7]. Thus, in the context of the plastid gene expression defects of the *prpl11*, *rif1* and *rif10* mutants, SP1 might help to efficiently down-regulate photosynthetic proteins by controlling protein import, acting in a way that is complementary to retrograde signalling. Defective plastid gene expression may compromise carbon fixation, e.g. due to insufficiency of Rubisco large subunit [S2], and this could lead to excessive ROS production. Our results show that failure to properly regulate protein import in these mutant backgrounds has adverse consequences for plants, possibly due to the accumulation of ROS. This indicates that protein import is finely regulated to cope with different states of chloroplast biogenesis.

Supplemental Experimental Procedures

Plant Growth Conditions and Physiological Studies

All *Arabidopsis thaliana* plants used in this work were of the Columbia-0 (Col-0) ecotype. The *sp1-1*, *sp1-2*, *sp1-3*, SP1 overexpressor (OX), *ppi1-1*, *enh1-2*, *sos2-2* and *sos3-1* mutant and transgenic lines have all been reported previously [S1, S8, S9, S10]. For in vitro growth, seeds were surface sterilized, sown on Murashige-Skoog (MS) agar medium in petri plates, cold-treated at 4°C, and thereafter kept in a growth chamber, as described previously [S11]. All plants were grown under a long-day cycle (16 hours light, 8 hours dark), except those used for protoplast isolation which were grown under short day conditions (8 hours light, 16 hours dark). Chlorophyll measurements were performed following *N*,*N'*-dimethylformamide (DMF) extraction using a spectrophotometer [S12, S13, S14].

For the stress experiments, all seeds of the different genotypes were harvested at the same time. Plants were either germinated directly on, or transferred from normal MS agar medium to, MS agar medium supplemented with 150 mM NaCl, 300-400 mM mannitol, or 1.0-1.3 µM paraquat (added before pouring the plates) for salt, osmotic or oxidative stress treatment, respectively. In general, germinating directly on stress medium was used to assess the stress phenotype, while transfer to stress medium was used to study the mechanism of stress tolerance (to avoid possible non-specific effects caused by phenotypical differences between genotypes). Sucrose was omitted from the MS medium in all stress experiments, except for those involving direct germination on NaCl plates (which contained 0.5% sucrose), to minimize possible non-specific effects [S15]. For assessing growth performance or survival rate, at least three experiments were performed, and ~50-300 seedlings per genotype were analysed in each experiment.

For Figures 1A-1C, plants were germinated on medium containing 150 mM NaCl and allowed to grow under standard conditions for 14 days prior to photography and scoring for a measure of stress tolerance and germination efficiency; stress tolerance was estimated by counting the number of green individuals per genotype, and expressing these numbers as a percentage of the total number of germinated plants per genotype. For Figure 1D, plants were germinated on medium containing 400 mM mannitol and allowed to grow for 3 weeks prior to scoring for a measure of stress tolerance; stress tolerance was estimated by counting the number of plants that had developed to the point of primary leaf emergence, and expressing these numbers as a percentage of the total number of plants per genotype (germination efficiency was essentially 100% in all genotypes; data not shown). For Figures 1E and 1F, plants were germinated on medium containing 1.3 µM paraquat and allowed to grow for 10 days prior to photography and scoring for death or survival as a measure

of stress sensitivity; dead plants were very small and white, whereas surviving plants had expanded, green cotyledons (germination efficiency was essentially 100% in all genotypes; data not shown).

For Figures 3A and 3B, plants were germinated on standard medium and allowed to grow for 7 days before transferral to medium containing 300 mM mannitol (or medium lacking mannitol as a control). The transferred plants were kept under standard conditions for a further 2 days prior to analysis. This short-term osmotic stress treatment was designed to enable detection of the primary effects of the stress upon analysis by immunoblotting.

Generation and Analysis of Double Mutants

Double homozygous mutants combining *sp1-3* with *enh1-2*, *sos2-2* or *sos3-1* were identified in the F2 or F3 generation of the corresponding crosses by PCR testing. The *sp1-3* mutation was identified by gene-specific primers 5'-GCTGTCAAGGACGATATTG-3' and 5'-CAGACAAGACTCAAGTAGAGAATG-3', and the former in combination with the left border T-DNA-specific primer 5'-GCGTGGACCGCTTGCTGCAACT-3'. The *enh1-2* and *sos3-1* mutations were identified as described previously [S9, S10]. The *sos2-2* mutation was identified by dCAPS analysis involving PCR with primers 5'-TTGGATGATATTCGTGCAGATC-3' and 5'-TTAACATTTAAATGGAATTGACC-3' followed by digestion with restriction enzyme *Bgl*II, and verified by DNA sequencing. The corresponding single homozygous mutants were also identified, as siblings in the same generations, and these were employed as controls for the phenotypical comparisons. For Figures 2C and 2D, plants were subjected to moderate oxidative stress by germination and growth for 7 days on medium containing 1 µM paraquat; at the end of the treatment period, the plants were photographed or subjected to chlorophyll content analysis. Enhanced phenotype severity in the *sp1 sos2* and *sp1 enh1* double mutants, but not in *sp1 sos3*, revealed synergistic genetic interactions and implied involvement of SP1 in ROS regulation like SOS2 and ENH1, but via a different mechanism.

ROS Detection

The detection of hydrogen peroxide was performed by staining with 3,3'-diaminobenzidine (DAB) (Sigma-Aldrich) using a modified published method [S16]. Briefly, seedlings growing on normal MS medium for 3 (for NaCl stress) or 8 (for mannitol and paraquat stresses) days (under these conditions, seedlings of all the genotypes appeared normal and developed similarly) were transferred to corresponding stress plates for 3 weeks (for NaCl stress), 3 days (for mannitol stress), or 2 days (for paraquat stress). Then, the shoots of the seedlings were collected and infiltrated for 10

minutes using a gentle vacuum with freshly-made staining solution (1 mg/mL DAB, 0.05% (v/v) Tween 20, and 10 mM sodium phosphate buffer, pH 7.0). For each experiment, 5-10 seedlings were used and the experiment was repeated at least twice. Infiltrated samples were incubated in the dark at room temperature with gentle shaking for 6 hours to overnight. The staining reaction was then terminated by replacing the staining solution with bleaching solution (ethanol:glycerol:acetic acid, 3:1:1 by volume) and incubation in a water bath at 95°C for 15 min. Then, the samples were incubated with fresh bleaching solution at room temperature until the chlorophyll was completely depleted, and transferred to ethanol:glycerol (4:1 by volume) for photography.

For Figures 2A and 2B, 8-day-old plants germinated on standard medium were transferred to medium containing 1 µM paraquat (or medium lacking paraquat as a control) and analysed after a further 2 days; such treatment generated moderate levels of oxidative stress without presenting survival issues, did not cause visibly different stress phenotypes in the different genotypes, and was found to be optimal for this analysis. For quantification, ImageJ was used to establish a threshold of DAB staining over the background, and to calculate the area of DAB staining versus the total area of the shoot.

Semi-Quantitative and Quantitative RT-PCR

RNA extraction, reverse transcription, and semi-quantitative or quantitative RT-PCR were performed as described previously [S17]. The primers used for PCR amplification were: *ACTIN*, 5'-CAAGCAGCATGAAGATTAAGGTCGTT-3' and 5'-CTTGGAGATCCACATCTGCTGGAAT-3'; *TOC33*, 5'-AATGGTGAAGCGTGGATC-3' and 5'-TGCTCCTTGAATCATCTTAACG-3'; *TOC159*, 5'-AACTCTTGAAGTGGCTAATAAGT-3' and 5'-ACAACCTCTGGCTCTACA-3'; and *TOC75*, 5'-TCGCATCTCCACTCAATC-3' and 5'-GTCTCTGTATCTCGGTTAGG-3'; and *CFP*, 5'-TACAACTACAACAGCCACAA-3' and 5'-CGGATCTTGAAGTTCACCTT-3'. Gene expression data were normalized using the data for *ACTIN*. To exclude the possibility of DNA contamination (genomic or transfected plasmid) in the RNA samples, which was especially important for the analysis presented in Figure 4F, appropriate amounts of RNA (equivalent to those used for cDNA synthesis) were similarly tested in control PCR experiments. No significant amplification signal was detected, eliminating the possibility of DNA contamination that might affect quantification.

Plasmid Construction and Protoplast Transient Assays

For the construct of OE33tp-CFP, the oxygen evolving complex 33 kD subunit (OE33; At5g66570) transit peptide was amplified by PCR from Col-0 cDNA, and then cloned into p2GWC7 [S18] using Gateway technology to produce a C-terminal fusion to CFP. OE33 has a bipartite targeting signal comprising a transit peptide of 29 amino acids followed by a thylakoid lumen targeting signal of 56 residues. Sequence encoding the whole transit peptide and part of the lumen targeting domain (37 amino acids) was amplified, to enable targeting of CFP protein into the chloroplast stroma in protoplasts. The YFP-HA construct has been described previously [S1].

Protoplast isolation and transient assays were carried out as described previously [S19], except that a culture buffer (500 mM mannitol, 4 mM 4-morpholineethanesulfonic acid [MES], pH 5.6, and 10 mM KCl) was used to replace W5 buffer for the final overnight incubation, to facilitate the following protein extraction. Protoplasts were isolated from plants grown under short day condition for 7-8 weeks. For either XFP fluorescence or immunoblotting assays, 0.1 mL (10^5) of protoplasts were transfected with 5 µg DNA. After incubation in the dark after 15 hours, the protoplasts were analysed, either under a microscope for XFP fluorescence or by immunoblotting. For immunoblotting assays, protoplasts were pelleted at 100g for 2 minutes and the pellet was added directly to 2× SDS-PAGE loading buffer (50 mM Tris-HCl, pH 6.8, 20% glycerol, 1% sodium dodecyl sulphate [SDS], and 0.1 M dithiothreitol), followed by SDS-PAGE and immunoblotting.

For Figures 4C-4H, protoplasts isolated from rosette leaves of wild-type, *sp1-3* and SP1 overexpressor plants were transfected with plasmids encoding either one or the other of the following chimeric proteins: transit peptide of OE33 fused to CFP (OE33tp-CFP), and YFP fused to the haemagglutinin tag (YFP-HA) as a control. Transfected protoplasts were analysed by immunoblotting to detect the transiently expressed protein as well as native Toc75 or Tic110. Specific bands detected in repeated experiments were quantified, and the values for each protein were normalized relative to corresponding Tic110 values, prior to further normalization relative to the wild type. For Figure 4F, RNA was extracted from OE33tp-CFP-transfected protoplasts (three independent samples per genotype), and subjected to quantitative RT-PCR analysis using *CFP*- and *ACTIN*-specific primers. The *CFP* data were normalized relative to corresponding *ACTIN* data, prior to further normalization relative to the wild type.

In Vitro Translation, Chloroplast Isolation, and Import

Previously described clones carrying the full-length cDNAs of OE33 (119E10T7) or PsaD (307C9T7) were employed to amplify template using M13 primers [S20]. The in vitro transcription/translation procedure was described previously [S13, S21]. For normal conditions, chloroplasts were isolated from 10-day-old plants grown in vitro on standard MS agar medium. For stress conditions, chloroplasts were isolated from plants that had been grown on standard MS agar medium for 8 days and then transferred into liquid MS medium supplemented with 200 mM mannitol for 2 days (under these conditions, we found the yield of chloroplasts and the degree of stress to be optimal for import assays; more severe treatments adversely affected yield and/or import competence of the organelles). Isolations and protein import were performed as described previously [S11, S22]. Thermolysin treatments were conducted using standard procedures [S1, S23]. Band intensities were quantified using Aida software (Raytest).

For Figures 4A and 4B, chloroplasts isolated from osmotically-stressed 10-day-old wild-type, *sp1-3* and SP1 overexpressor seedlings (normalized according to chlorophyll amount) were incubated with in vitro translated, radiolabelled precursors of OE33 and PsaD under import conditions. The final time-point in each case was conducted in duplicate, with one sample being treated with thermolysin protease to remove un-imported precursor. Bands corresponding to the intermediate and mature forms of OE33, and to the mature form of PsaD, were quantified, and the values were normalized relative to those obtained for wild-type chloroplasts at the final time-point.

Immunoblotting

Immunoblotting was performed as described previously [S24, S25] with minor modifications. Primary antibodies were anti-Toc75-III [S17], anti-atToc33 peptide antibody [S26], anti-atTic40 [S17], anti-atTic110 [S27], anti-atToc159 [S28], anti-OEP80 [S29], anti-OE33 [S1], anti-PsaD [S30], anti-HA (Sigma), and anti-YFP (BD Biosciences). Secondary antibodies were anti-rabbit IgG conjugated with either alkaline phosphatase (Sigma) or horseradish peroxidase (Santa Cruz Biotechnology). Chemiluminescence was detected using ECL Plus Western Blotting Detection Reagent (GE Healthcare) and an LAS-4000 imager (Fujifilm, GE Healthcare). Band intensities were quantified using Aida software (Raytest).

For Figures 3A and 3B, specific bands detected by immunoblotting in four similar experiments were quantified, and the values obtained were normalized relative to corresponding data for the

Coomassie-stained Rubisco large subunit band; data for each genotype were normalized relative to the wild type.

Confocal Laser-Scanning Microscopy

For the imaging of CFP, YFP and chlorophyll fluorescence signals, protoplasts were examined using a Zeiss LSM 510 META laser-scanning microscope (Carl Zeiss Ltd., Welwyn Garden City, Herts, UK). The CFP fluorescence was excited with a 458-nm excitation line of a 25 mW argon ion laser and an HFT 458/514 primary dichroic mirror, and was detected by a 475- to 525-nm band-pass filter in the single-track facility of the microscope. To detect YFP, a 514-nm excitation from a 5 mW argon ion laser with an HFT 458/514 primary dichroic mirror and a 535- to 590-nm emission filter was used. In each case, to simultaneously detect chlorophyll autofluorescence, a NFT 635 vis long-pass filter was used. Images were processed using Zeiss LSM Image Browser. All experiments were conducted at least twice with the same results, and typical images are shown.

Underlying research materials are available from the corresponding author upon request.

Supplemental References

S1. Ling, Q., Huang, W., Baldwin, A., and Jarvis, P. (2012). Chloroplast biogenesis is regulated by direct action of the ubiquitin-proteasome system. Science *338*, 655-659.

S2. Pesaresi, P., Varotto, C., Meurer, J., Jahns, P., Salamini, F., and Leister, D. (2001). Knock-out of the plastid ribosomal protein L11 in *Arabidopsis*: effects on mRNA translation and photosynthesis. Plant J. *27*, 179-189.

S3. Flores-Perez, U., Sauret-Gueto, S., Gas, E., Jarvis, P., and Rodriguez-Concepcion, M. (2008). A mutant impaired in the production of plastome-encoded proteins uncovers a mechanism for the homeostasis of isoprenoid biosynthetic enzymes in Arabidopsis plastids. Plant Cell *20*, 1303-1315.

S4. Sauret-Güeto, S., Botella-Pavía, P., Flores-Pérez, U., Martínez-García, J.F., San Román, C., León, P., Boronat, A., and Rodríguez-Concepción, M. (2006). Plastid cues posttranscriptionally regulate the accumulation of key enzymes of the methylerythritol phosphate pathway in Arabidopsis. Plant Physiol. *141*, 75-84.

S5. Koussevitzky, S., Nott, A., Mockler, T.C., Hong, F., Sachetto-Martins, G., Surpin, M., Lim, J., Mittler, R., and Chory, J. (2007). Signals from chloroplasts converge to regulate nuclear gene expression. Science *316*, 715-719.

S6. Sun, X., Feng, P., Xu, X., Guo, H., Ma, J., Chi, W., Lin, R., Lu, C., and Zhang, L. (2011). A chloroplast envelope-bound PHD transcription factor mediates chloroplast signals to the nucleus. Nat. Commun. *2*, 477.

S7. Pesaresi, P., Masiero, S., Eubel, H., Braun, H.P., Bhushan, S., Glaser, E., Salamini, F., and Leister, D. (2006). Nuclear photosynthetic gene expression is synergistically modulated by rates of protein synthesis in chloroplasts and mitochondria. Plant Cell *18*, 970-991.

S8. Liu, J., Ishitani, M., Halfter, U., Kim, C.S., and Zhu, J.K. (2000). The *Arabidopsis thaliana SOS2* gene encodes a protein kinase that is required for salt tolerance. Proc. Natl. Acad. Sci. USA *97*, 3730-3734.

S9. Halfter, U., Ishitani, M., and Zhu, J.K. (2000). The *Arabidopsis* SOS2 protein kinase physically interacts with and is activated by the calcium-binding protein SOS3. Proc. Natl. Acad. Sci. USA *97*, 3735-3740.

S10. Zhu, J., Fu, X., Koo, Y.D., Zhu, J.K., Jenney, F.E., Jr., Adams, M.W., Zhu, Y., Shi, H., Yun, D.J., Hasegawa, P.M., et al. (2007). An enhancer mutant of *Arabidopsis salt overly sensitive 3* mediates both ion homeostasis and the oxidative stress response. Mol. Cell. Biol. *27*, 5214-5224.

S11. Aronsson, H., and Jarvis, P. (2002). A simple method for isolating import-competent *Arabidopsis* chloroplasts. FEBS Lett. *529*, 215-220.

S12. Jarvis, P., Chen, L.J., Li, H., Peto, C.A., Fankhauser, C., and Chory, J. (1998). An *Arabidopsis* mutant defective in the plastid general protein import apparatus. Science *282*, 100-103.

S13. Huang, W., Ling, Q., Bédard, J., Lilley, K., and Jarvis, P. (2011). In vivo analyses of the roles of essential Omp85-related proteins in the chloroplast outer envelope membrane. Plant Physiol. *157*, 147-159.

S14. Constan, D., Patel, R., Keegstra, K., and Jarvis, P. (2004). An outer envelope membrane component of the plastid protein import apparatus plays an essential role in *Arabidopsis*. Plant J. *38*, 93-106.

S15. Verslues, P.E., Agarwal, M., Katiyar-Agarwal, S., Zhu, J., and Zhu, J.K. (2006). Methods and concepts in quantifying resistance to drought, salt and freezing, abiotic stresses that affect plant water status. Plant J. *45*, 523-539.

S16. Daudi, A., Cheng, Z., O'Brien, J.A., Mammarella, N., Khan, S., Ausubel, F.M., and Bolwell, G.P. (2012). The apoplastic oxidative burst peroxidase in *Arabidopsis* is a major component of pattern-triggered immunity. Plant Cell *24*, 275-287.

S17. Kasmati, A.R., Töpel, M., Patel, R., Murtaza, G., and Jarvis, P. (2011). Molecular and genetic analyses of Tic20 homologues in *Arabidopsis thaliana* chloroplasts. Plant J. *66*, 877-889.
S18. Karimi, M., De Meyer, B., and Hilson, P. (2005). Modular cloning in plant cells. Trends Plant Sci. *10*, 103-105.
S19. Wu, F.H., Shen, S.C., Lee, L.Y., Lee, S.H., Chan, M.T., and Lin, C.S. (2009). Tape-Arabidopsis Sandwich - a simpler Arabidopsis protoplast isolation method. Plant Methods *5*, 16.
S20. Kubis, S., Baldwin, A., Patel, R., Razzaq, A., Dupree, P., Lilley, K., Kurth, J., Leister, D., and Jarvis, P. (2003). The Arabidopsis *ppi1* mutant is specifically defective in the expression, chloroplast import, and accumulation of photosynthetic proteins. Plant Cell *15*, 1859-1871.
S21. Baldwin, A., Wardle, A., Patel, R., Dudley, P., Park, S.K., Twell, D., Inoue, K., and Jarvis, P. (2005). A molecular-genetic study of the Arabidopsis Toc75 gene family. Plant Physiol. *138*, 715-733.
S22. Aronsson, H., and Jarvis, R.P. (2011). Rapid isolation of Arabidopsis chloroplasts and their use for in vitro protein import assays. Methods Mol. Biol. *774*, 281-305.
S23. Froehlich, J. (2011). Studying Arabidopsis envelope protein localization and topology using thermolysin and trypsin proteases. Methods Mol. Biol. *774*, 351-367.
S24. Kovacheva, S., Bédard, J., Patel, R., Dudley, P., Twell, D., Ríos, G., Koncz, C., and Jarvis, P. (2005). *In vivo* studies on the roles of Tic110, Tic40 and Hsp93 during chloroplast protein import. Plant J. *41*, 412-428.
S25. Kovacheva, S., Bédard, J., Wardle, A., Patel, R., and Jarvis, P. (2007). Further *in vivo* studies on the role of the molecular chaperone, Hsp93, in plastid protein import. Plant J. *50*, 364-379.
S26. Aronsson, H., Combe, J., and Jarvis, P. (2003). Unusual nucleotide-binding properties of the chloroplast protein import receptor, atToc33. FEBS Lett. *544*, 79-85.
S27. Aronsson, H., Combe, J., Patel, R., Agne, B., Martin, M., Kessler, F., and Jarvis, P. (2010). Nucleotide binding and dimerization at the chloroplast pre-protein import receptor, atToc33, are not essential *in vivo* but do increase import efficiency. Plant J. *63*, 297-311.
S28. Bauer, J., Chen, K., Hiltbrunner, A., Wehrli, E., Eugster, M., Schnell, D., and Kessler, F. (2000). The major protein import receptor of plastids is essential for chloroplast biogenesis. Nature *403*, 203-207.
S29. Hsu, S.C., Nafati, M., and Inoue, K. (2012). OEP80, an essential protein paralogous to the chloroplast protein translocation channel Toc75, exists as a 70-kD protein in the *Arabidopsis thaliana* chloroplast outer envelope. Plant Mol. Biol. *78*, 147-158.
S30. Andersen, B., Koch, B., and Scheller, H.V. (1992). Structural and functional analysis of the reducing side of photosystem I. Physiol. Plant. *84*, 154-161.
