# Supplementary material for: Regulation of Chloroplast Protein Import by the Ubiquitin E3 Ligase SP1 Is Important for Stress Tolerance in Plants
Source: Curr Biol. 2015 Oct 5;25(19):2527–34. doi: 10.1016/j.cub.2015.08.015 (PMC4598742; doi:10.1016/j.cub.2015.08.015)
Supplement: Document S2. Article plus Supplemental Information [file mmc2.pdf]

Report

Current Biology

Regulation of Chloroplast Protein Import by the Ubiquitin E3 Ligase SP1 Is Important for Stress Tolerance in Plants

Graphical Abstract

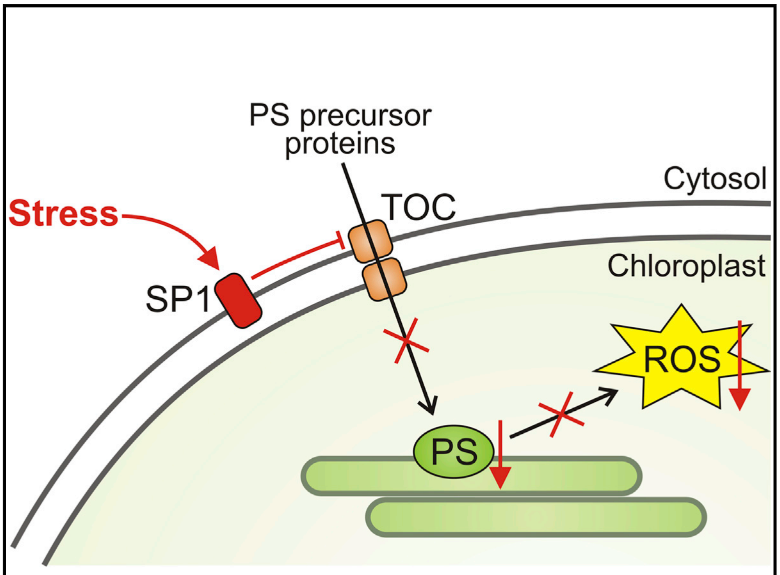

Highlights

- Levels of the chloroplast E3 ligase SP1 influence plant abiotic stress tolerance
- Effects of SP1 on stress tolerance are linked to reactive oxygen species levels
- SP1 acts to deplete the chloroplast protein import (TOC) machinery under stress
- TOC depletion by SP1 is linked to reduced plastid import of photosynthesis proteins

Authors

Qihua Ling, Paul Jarvis

Correspondence

paul.jarvis@plants.ox.ac.uk

In Brief

A harmful effect of abiotic stress in plants is photo-oxidation linked to overproduction of reactive oxygen species (ROS) by the photosynthetic machinery. Ling and Jarvis show that the E3 ligase SP1 promotes stress tolerance by depleting the chloroplast protein import apparatus, which limits photosystem assembly and the potential for ROS formation.

Ling & Jarvis, 2015, Current Biology *25*, 2527–2534
October 5, 2015 ©2015 The Authors
http://dx.doi.org/10.1016/j.cub.2015.08.015

Current Biology
Report

Regulation of Chloroplast Protein Import by the Ubiquitin E3 Ligase SP1 Is Important for Stress Tolerance in Plants

Qihua Ling[1] and Paul Jarvis[1,*]
[1]Department of Plant Sciences, University of Oxford, South Parks Road, Oxford OX1 3RB, UK
*Correspondence: paul.jarvis@plants.ox.ac.uk
http://dx.doi.org/10.1016/j.cub.2015.08.015
This is an open access article under the CC BY license (http://creativecommons.org/licenses/by/4.0/).

SUMMARY

Chloroplasts are the organelles responsible for photosynthesis in plants [1, 2]. The chloroplast proteome comprises ~3,000 different proteins, including components of the photosynthetic apparatus, which are highly abundant. Most chloroplast proteins are nucleus-encoded and imported following synthesis in the cytosol. Such import is mediated by multiprotein complexes in the envelope membranes that surround each organelle [3, 4]. The translocon at the outer envelope membrane of chloroplasts (TOC) mediates client protein recognition and early stages of import. The TOC apparatus is regulated by the ubiquitin-proteasome system (UPS) in a process controlled by the envelope-localized ubiquitin E3 ligase SUPPRESSOR OF PPI1 LOCUS1 (SP1) [5, 6]. Previous work showed that SP1-mediated regulation of chloroplast protein import contributes to the organellar proteome changes that occur during plant development (e.g., during de-etiolation). Here, we reveal a critical role for SP1 in plant responses to abiotic stress, which is a major and increasing cause of agricultural yield losses globally [7]. *Arabidopsis* plants lacking SP1 are hypersensitive to salt, osmotic, and oxidative stresses, whereas plants overexpressing SP1 are considerably more stress tolerant than wild-type. We present evidence that SP1 acts to deplete the TOC apparatus under stress conditions to limit the import of photosynthetic apparatus components, which may attenuate photosynthetic activity and reduce the potential for reactive oxygen species production and photo-oxidative damage. Our results indicate that chloroplast protein import is responsive to environmental cues, enabling dynamic regulation of the organellar proteome, and suggest new approaches for improving stress tolerance in crops.

RESULTS AND DISCUSSION

SP1 Expression Levels Influence Abiotic Stress Tolerance

Because SUPPRESSOR OF PPI1 LOCUS1 (SP1) is an important mediator of the chloroplast protein import and proteome changes that occur during plant development [5], we wished to investigate whether this E3 ligase is similarly involved in those changes that occur during plant responses to abiotic stress. This possibility was suggested by reports showing that changes in photosynthetic activity and the chloroplast proteome form an important component of plant stress responses [8, 9]. Thus, we grew *sp1* mutant and SP1 overexpressor (OX) *Arabidopsis* plants under different abiotic stress conditions, starting with high salinity (150 mM NaCl). Mutant plants failed to develop under these conditions, whereas SP1 overexpressors were more stress tolerant than wild-type (Figures 1A and 1B); in neither case could the developmental and greening differences be accounted for by differences in germination efficiency (Figure 1C). Similar results were obtained in relation to osmotic stress (300–400 mM mannitol), using both primary leaf emergence and chlorophyll accumulation as measures of stress tolerance (Figures 1D and S1A–S1C). By contrast, the different genotypes were indistinguishable when grown on normal medium under the same growth conditions (Figure S1D).

An important component of salinity and osmotic stresses is oxidative stress, linked to the overproduction of reactive oxygen species (ROS), which can damage cellular components. Because SP1 resides in chloroplasts, an important source of ROS [8, 9], we suspected that SP1's role in stress is linked to oxidative effects. To assess this possibility, we subjected the same plant genotypes to oxidative stress induced by the herbicide paraquat (1.3 μM), which interferes with photosynthetic electron transport. As expected, *sp1* mutants showed a higher death rate under these conditions, whereas SP1 overexpressors were more stress tolerant than wild-type (Figures 1E, 1F, and S1E).

Chloroplast protein import is impaired under temperature stress [10]. Thus, we tested the effect of temperature stress on *sp1* mutant and SP1 overexpressor plants, using the reported conditions. However, no obvious differences between the genotypes could be seen (data not shown). We also applied high-light stress using an established method [11], but again, no clear differences between the genotypes were found (data not shown).

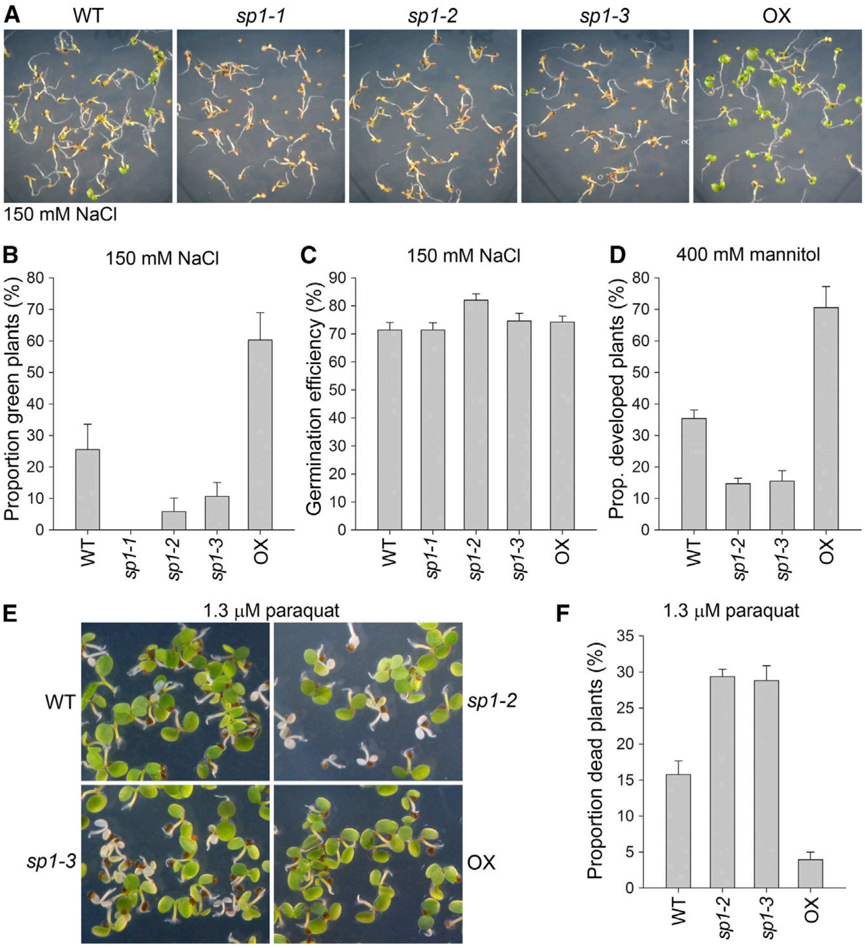

Figure 1. SP1 Expression Levels Influence Abiotic Stress Tolerance

(A–C) Plants grown under high-salinity stress were photographed (A) and scored for a measure of stress tolerance (B) and germination efficiency (C). Error bars indicate SEM (n = 3).

(D) Plants grown under osmotic stress were scored for a measure of stress tolerance. Error bars indicate SEM (n = 6).

(E and F) Plants grown under oxidative stress were photographed (E) and scored for death/survival as a measure of stress sensitivity (F). Error bars indicate SEM (n = 3).

These results suggest (1) that SP1 is involved in the tolerance of some, but not all, abiotic stresses, (2) that redundant pathways may compensate for the loss of SP1 under certain conditions, or (3) that some stresses, e.g., extreme light, might have consequences so severe or direct that they overwhelm the SP1-dependent mechanism, rendering it unable to cope. The latter two possibilities seem more likely, as *SP1* transcript levels are elevated under various abiotic stresses including not only drought and osmotic stresses but also temperature and light intensity stresses [12]. SP1 may also act in biotic stress, although the mechanisms involved are unknown [13].

Effects of SP1 on Stress Tolerance Are Linked to ROS Regulation

Accumulation of the purple pigment anthocyanin occurred under salt stress in all genotypes except the SP1 overexpressor (Figure S2A). As anthocyanin acts as an antioxidant and is a sign of ROS overproduction [14], we hypothesized that SP1's role under stress is to control ROS levels. To directly assess whether the effects of SP1 on stress tolerance are linked to ROS, we stained stressed plants with 3,3′-diaminobenzidine (DAB), which detects hydrogen peroxide, a stable and frequently analyzed ROS molecule [15]. Under all three stress conditions (salinity, osmotic, and oxidative), we observed enhanced DAB staining in *sp1* mutants and reduced staining in SP1 overexpressor plants, relative to wild-type (Figures 2A, 2B, S2B, and S2C). In fact, stressed SP1 overexpressors were indistinguishable from unstressed control plants in relation to DAB staining (Figures 2A and 2B), implying that SP1 overexpression provides a high level of stress tolerance. Thus, the stress tolerance effect of SP1 is inversely correlated with levels of an important ROS.

Previously, *sp1* mutant and SP1 overexpressor plants were shown to exhibit plastid-linked developmental differences under certain challenging conditions (applied for the induction of de-etiolation and leaf senescence) [5]. This raises a question about whether the stress sensitivity and ROS accumulation differences seen here were due to inherent developmental differences between the genotypes. However, mutant and overexpressor plants displayed no obvious growth defects or ROS accumulation differences under the normal (unchallenging) growth conditions employed here (Figures S1D and 2A). Thus, the observed SP1-dependent stress effects were indeed stress-specific responses and unlikely to be the result of intrinsic growth differences between the genotypes.

To further investigate the link between SP1 and oxidative stress, we crossed *sp1* to three well-characterized salt-sensitive mutants, two of which have effects linked to ROS, and identified the corresponding double mutants [16, 17]. The *salt overly sensitive2* (*sos2*) mutation affects a kinase that controls activity of the plasma membrane Na^+/H^+ antiporter SOS1 and has an additional role in ROS regulation, whereas *enhancer1 of sos3* (*enh1*) affects a chloroplast protein with possible electron carrier function in ROS detoxification. The *sos3* mutation also affects SOS1 activity, in response to cytosolic calcium, but unlike *sos2*, its role seems to be restricted to ion homeostasis. Genetic analyses imply that the SOS2 and ENH1 proteins work in the same pathway of ROS regulation [17]. In accordance with our preceding results, the *sp1 sos2* and *sp1 enh1* double mutants were even more sensitive to oxidative stress than the already sensitive *sos2* and *enh1* single mutants (Figures 2C and 2D). Enhanced sensitivity of the double mutants cannot be attributed to simple phenotype additivity, as *sp1* single mutants

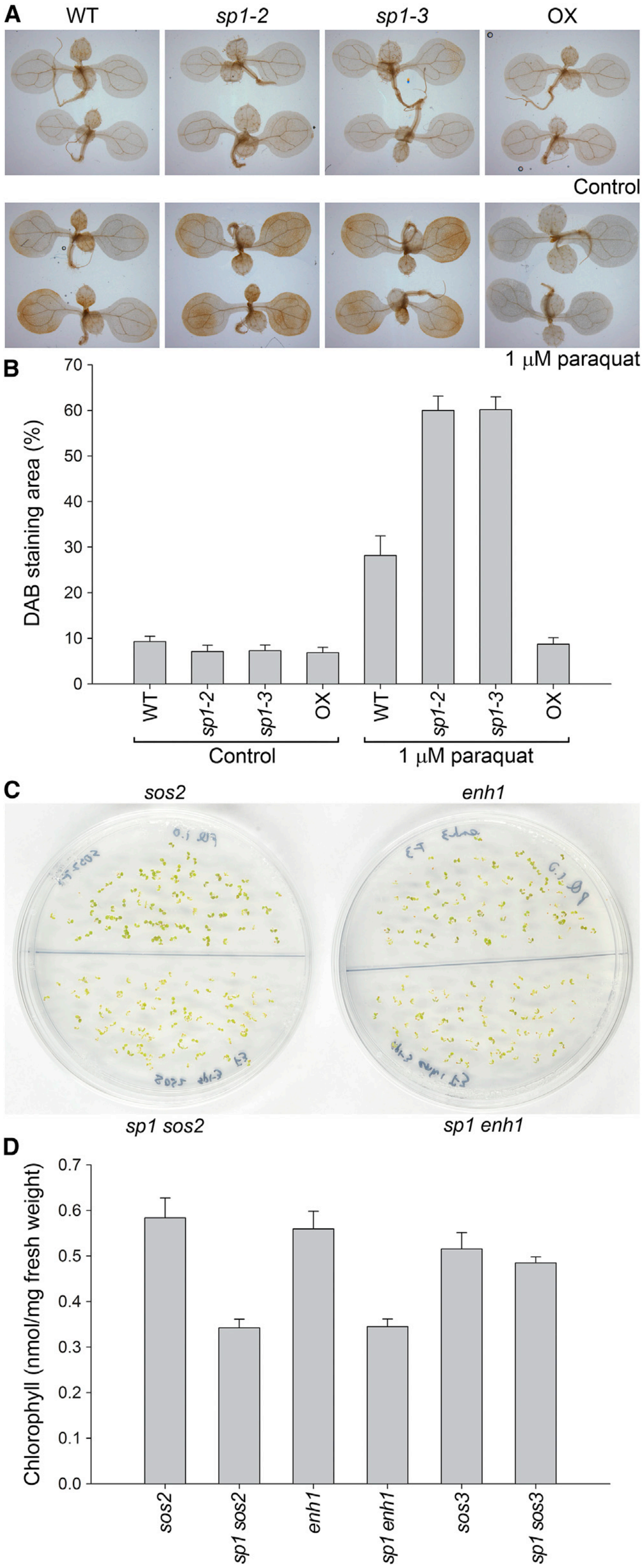

Figure 2. Effects of SP1 on Stress Tolerance Are Linked to ROS

(A and B) Staining for hydrogen peroxide accumulation following oxidative stress. Stressed and control, mock-treated plants were stained with 3,3′-diaminobenzidine (DAB), and representative images are shown (A). The area of intense brown staining (indicative of H_2O_2 accumulation) was quantified and expressed as a percentage of the total surface area for each plant (B). Error bars indicate SEM (n = 10).

(C and D) Genetic interactions between *sp1* and the salt sensitivity mutations *sos2*, *enh1*, and *sos3*. Plants subjected to moderate oxidative stress were photographed (C) or subjected to chlorophyll content analysis as a measure of phenotype severity (D). Error bars indicate SEM (n = 3).

were indistinguishable from wild-type under the moderate stress conditions employed (1 μM paraquat; Figures S2D and S2E). By contrast, *sp1 sos3* double mutants were not significantly more sensitive to oxidative stress than *sos3* (Figure 2D). Synergistic interactions similar to those seen here between *sp1* and either *sos2* or *enh1* are normally observed when two key components of the same pathway are both defective [18], and so we conclude that the role of SP1 in abiotic stress responses is closely connected to ROS regulation and involves a different mechanism from SOS2 or ENH1. There are two general mechanisms whereby plants regulate ROS: scavenging and avoidance [19]. SOS2 and ENH1 are thought to act in ROS scavenging [17], and so SP1 might act in avoidance. Avoidance strategies serve to reduce the production of ROS, for example by repressing photosynthesis.

Effects of SP1 on Stress Tolerance Are Linked to the Chloroplast Protein Import Machinery

We wished to understand the molecular basis for the stress tolerance mediated by SP1. Because the primary function of SP1 is to control translocon at the outer envelope membrane of chloroplasts (TOC) protein abundance [5], we began by examining the levels of important components of the chloroplast protein import machinery, in *sp1* mutant and SP1 overexpressor plants, under stress conditions. For this work, we focused on moderate, short-term osmotic stress (established seedlings were transferred to 300 mM mannitol for 2 days), to ensure viability of the plants, to avoid strong morphological changes that might have pleiotropic consequences, and to aid identification of the primary effects of stress. The results revealed that TOC protein levels decline markedly in response to stress in the wild-type (Figures 3A and 3B). This response was SP1 dependent, as it did not occur in *sp1* mutants, whereas the TOC components reached even lower levels in SP1 overexpressor plants. In contrast with the TOC proteins, components of the translocon at the inner envelope membrane of chloroplasts (TIC) did not change in abundance in response to stress, nor did an outer membrane protein uninvolved in the protein import mechanism, outer envelope protein 80 (OEP80) [3, 4, 20] (Figures 3A and 3B). Moreover, inspection of the protein banding pattern following staining with Coomassie revealed that the effect on TOC proteins was unlikely to be a general, damage-related response affecting many proteins (Figure 3A). qRT-PCR analyses did not reveal significant changes in TOC transcript levels under the conditions employed, suggesting a post-translational effect, which is consistent with the aforementioned dependency of the protein changes on SP1 (Figures S3A and S3B).

Under the short-term stress conditions described above, the abundance of photosynthetic apparatus components was unchanged (data not shown). However, following more-prolonged stress treatment, the abundance of such proteins declined markedly and the magnitude of this response was SP1 dependent

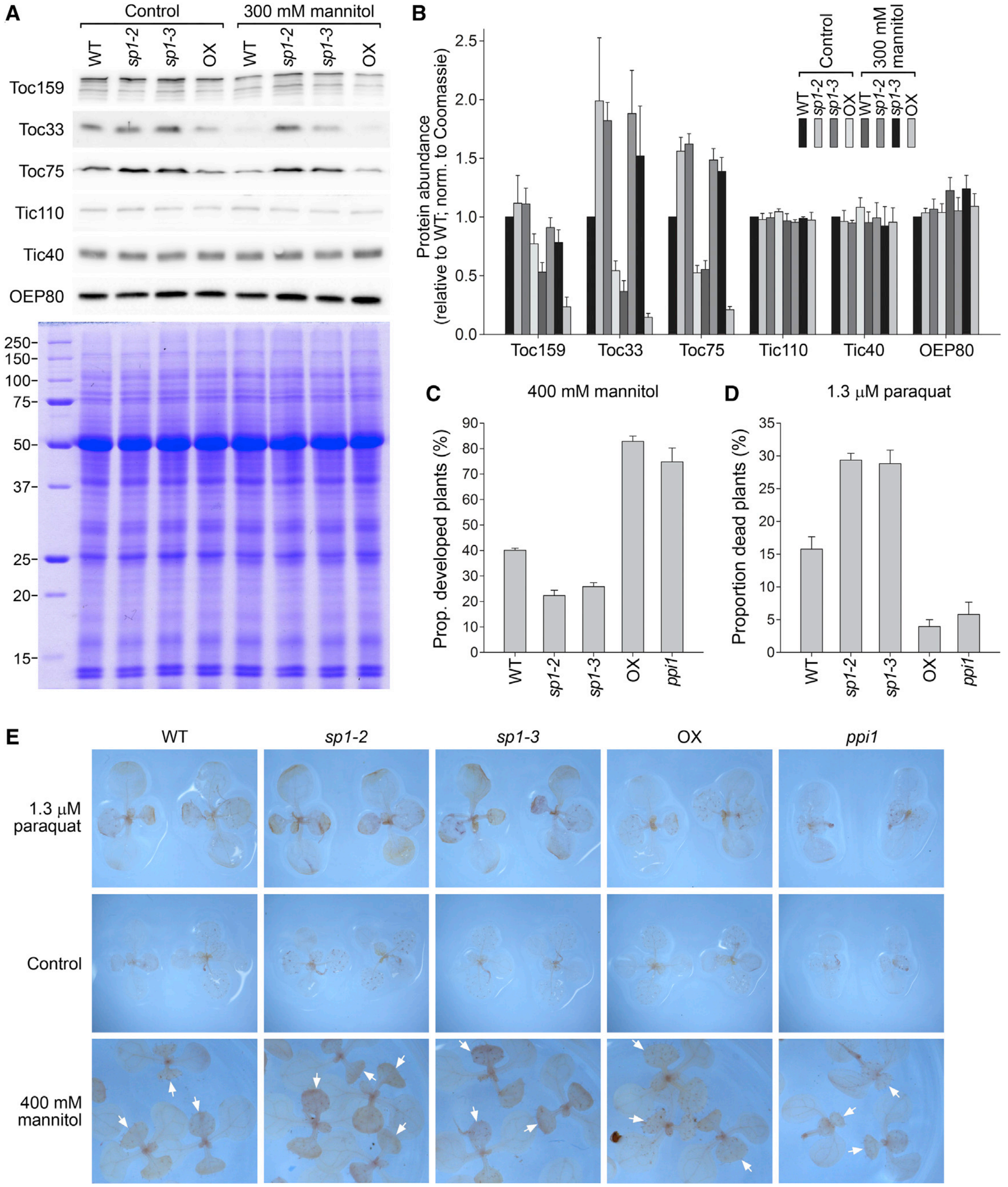

Figure 3. Effects of SP1 on Stress Tolerance Are Linked to the Chloroplast Protein Import Machinery

(A and B) Response of TOC protein levels to short-term osmotic stress. Total protein extracts from stressed and control plant material were analyzed by immunoblotting and staining with Coomassie Brilliant Blue (A), and specific bands were quantified (B). Error bars indicate SEM (n = 4).

(C–E) Abiotic stress tolerance of the chloroplast protein import mutant *ppi1*. Analyses of osmotic and oxidative stress responses were conducted as in Figure 1. Error bars indicate SEM (n = 3 or 4). Stressed and control, mock-treated plants were stained with DAB, and representative images are shown (E). Following osmotic stress, DAB staining occurred mainly in the true leaves (see arrows).

(Figures S3C and S3D). Thus, the data suggested a hypothesis in which SP1 acts to deplete the TOC apparatus under stress in order to limit the import of components of the photosynthetic machinery. It is well known that photosynthesis-related genes are transcriptionally downregulated under stress [9]. These two aspects of regulation (i.e., transcriptional and post-translational) might have the same eventual consequence of attenuated photosynthesis, reducing the potential for ROS overproduction and photo-oxidative damage, thereby promoting stress tolerance [21, 22]. Suppression of nuclear photosynthetic genes is mediated by retrograde chloroplast-to-nucleus signals [1], and this might only be efficient over the long term. By contrast,

SP1-mediated regulation would occur directly at the outer envelope (an ideal position facing both the cytosol and the chloroplast) [5], rapidly initiating responses to changes in the cytosolic and/or chloroplast environments post-translationally during stress. This would explain why TOC proteins are more quickly depleted than other chloroplast components.

The above hypothesis predicts that a TOC mutant with reduced capacity to import photosynthesis-related proteins, such as *plastid protein import1* (*ppi1*) [23], would show abiotic stress tolerance similarly to SP1 overexpressor plants. To investigate this possibility, we grew *ppi1* plants under the osmotic and oxidative stress conditions used previously. In accordance with the hypothesis, *ppi1* plants showed similar levels of stress tolerance to SP1 overexpressors, under both conditions (Figures 3C and 3D). Moreover, in each case, DAB staining revealed ROS levels to be similarly low in *ppi1* mutant and SP1 overexpressor plants (Figure 3E).

Although *ppi1* mutant and SP1 overexpressor plants display similar degrees of stress tolerance, *ppi1* mutants are pale yellow and considerably less vigorous under normal conditions than green SP1 overexpressors [5, 24]. This may be explained as follows: TOC functionality is constitutively impaired in *ppi1* (a single core TOC component, Toc33, is permanently missing), whereas in SP1 overexpressor plants, the effect is more nuanced and more balanced across the different TOC components; SP1 overexpression may increase survival rates with a smaller overall sacrifice of photosynthetic performance. Thus, whereas TOC component knockout mutations such as *ppi1* are unlikely to find stress-related applications in agriculture, SP1 overexpression may be an effective strategy in generating crops better able to cope with abiotic stresses linked to climate change, soil salinification, and other anthropogenic effects [7, 25].

Elevated SP1 Levels Can Reduce the Import of Photosynthesis-Related Proteins

Implicit in the aforementioned hypothesis of import regulation as a component of plant stress response is a requirement that SP1 activity should be elevated under stress conditions. The SP1 protein is autoregulated [5], and its abundance is maintained at extremely low levels, so that we are hardly able to detect it by immunoblotting (data not shown). Nonetheless, *SP1* transcript levels are elevated under stress conditions [12], and we therefore assume that protein levels are similarly increased. Activity and/or stability of SP1 might also be regulated dynamically by post-translational modification, which is common for E3 ligases [26]. To test whether altered SP1 levels, and corresponding TOC protein changes, can indeed influence chloroplast protein import efficiency, we compared the import capabilities in vitro of chloroplasts isolated from wild-type, *sp1* mutant, and SP1 overexpressor plants kept under either normal or stress conditions. The chloroplasts were incubated with precursors of the 33-kD subunit of the oxygen evolving complex (OE33) of photosystem II and of subunit D of photosystem I (PsaD), and import was assessed by quantifying the amount of mature, processed protein in the organelles (the identity of which was confirmed by resistance to thermolysin protease treatment [27]) (Figures 4A, 4B, S4A and S4B). The results showed that import of both proteins into SP1 overexpressor chloroplasts was reduced, relative to wild-type, under both conditions. Although *sp1* mutant chloroplasts had import capacity similar to the wild-type in the absence of stress (Figures S4A and S4B), implying that in young seedlings under such conditions the loss of SP1 has minimal consequence, the importance of SP1 was clearly demonstrated following stress treatment as *sp1* mutant chloroplasts displayed elevated import efficiency under these conditions, relative to wild-type (Figures 4A and 4B). These data support the notion that changing SP1 activity acts to regulate the import of photosynthetic machinery components and thus influences stress tolerance. The differing performance of *sp1* mutant chloroplasts under the two conditions probably reflects the fact that TOC abundance differences between wild-type and *sp1* are much more pronounced under stress conditions (Figures 3A and 3B).

To corroborate in a cellular context the results obtained with isolated chloroplasts, we conducted in vivo import experiments based on the transient expression of a chimeric precursor protein (comprising the transit peptide of OE33 fused to CFP; OE33tp-CFP) in transfected protoplasts of each genotype. Chloroplast import of the transiently expressed protein was assessed by monitoring the amount of its mature, processed form by immunoblotting (Figures 4C and 4D). In agreement with the in vitro experiments, chloroplast import was significantly reduced in SP1 overexpressor cells relative to wild-type, providing further support for our hypothesis of SP1-mediated import regulation. In addition, *sp1* protoplasts displayed elevated levels of protein import, which is consistent with the in vitro assays conducted using stressed plants (as protoplastation and transfection are stressful, employing 500 mM mannitol, this is the more-appropriate comparison; see below).

Import capacity differences between the genotypes observed in vivo reflected differences in the abundance of a key TOC component, Toc75, which forms the central, protein-conducting channel (Figures 4C and 4E). This implies that SP1-dependent import regulation affects a broad spectrum of plastid proteins and not just photosynthesis-related proteins, as Toc75 is a general import channel. Thus, SP1-mediated regulation of import under stress conditions may influence other adaptive mechanisms that depend on plastid proteins [28, 29], in addition to photosynthesis. Incongruence of the in vivo and in vitro (normal conditions) import data sets for the *sp1* mutant may be explained in terms of TOC abundance differences, in turn linked to the stress of protoplastation and/or to the age of the leaf material employed (older rosette leaves were used in the transfection studies, whereas young seedlings were employed for chloroplast isolation). The *sp1* mutation has only a moderate effect on TOC levels in young seedlings growing under normal conditions, but as leaves age, TOC proteins gradually accumulate in the absence of SP1 activity [5] (Figures 4C and 4E), and this may be partly responsible for the elevated import capacity seen for *sp1* in the in vivo assay. The stress of protoplastation may also cause TOC protein abundance differences between *sp1* and wild-type in the in vivo assay (paralleling the effect seen in osmotically stressed plants; Figures 3A and 3B), making the in vitro import results for stressed plants a more-valid comparison. Overall, these data further confirm that TOC protein levels, controlled by SP1, are positively correlated with protein import efficiency.

Differences in the abundance of the transiently expressed protein between genotypes were not linked to transfection or expression inconsistencies between the genotypes, as the

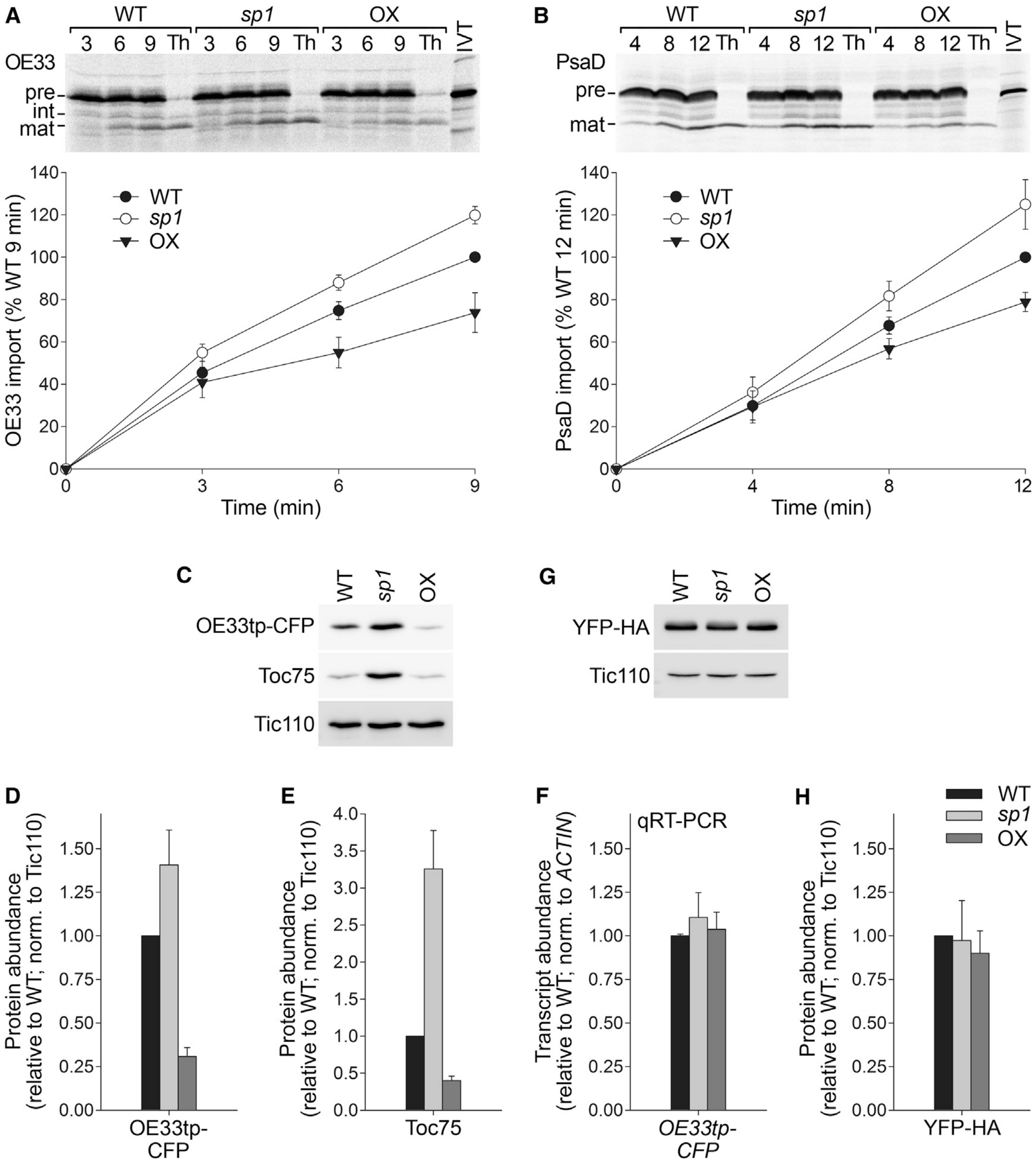

Figure 4. SP1 Influences the Import of Photosynthesis-Related Proteins

(A and B) In vitro protein import analysis using chloroplasts isolated from osmotically stressed plants. Import was allowed to proceed for the times shown, and then precursor (p), intermediate (i), and mature (m) protein forms associated with the chloroplasts were analyzed by SDS-PAGE and phosphorimaging. Representative images are shown. IVT, in vitro translation product used in each case; Th, thermolysin protease treatment. Bands corresponding to imported proteins were quantified. Error bars indicate SEM (n = 4 or 5).

(C–H) In vivo protein import analysis. Protoplasts were transfected with plasmids encoding either OE33tp-CFP or YFP fused to the hemagglutinin tag (YFP-HA) as a control. Immunoblotting was used to detect the transiently expressed proteins as well as native Toc75 or Tic110 (C and G). The band shown for OE33tp-CFP corresponds to the mature protein generated following transit peptide cleavage, as judged by size comparison with YFP-HA (data not shown). Specific bands were quantified (D, E, and H). In addition, RNA extracted from protoplast samples identical to those shown in (C) were subjected to qRT-PCR analysis using *CFP*- and *ACTIN*-specific primers (F). Error bars indicate SEM (n = 4 or 5 for D, E, and H; n = 3 for F).

expression level of *OE33tp-CFP* was similar in the different genotypes, as revealed by qRT-PCR (Figure 4F). In addition, in separate experiments, a control protein similar to the mature form of OE33tp-CFP, but not targeted to chloroplasts (YFP-HA), was expressed equally in all genotypes (Figures 4G and 4H). Analysis of transfected protoplasts by confocal microscopy confirmed that OE33tp-CFP was targeted to chloroplasts in all three genotypes, with efficiencies concordant with those seen by immunoblotting, and that YFP-HA remained in the cytosol (Figures S4C and S4D).

The data indicate that SP1 function is particularly important under challenging conditions when the optimization of import rates becomes critical. Such circumstances may include external stress, as elaborated above, or endogenous defects in chloroplast biogenesis (Figures S4E and S4F).

Conclusions

Our results provide a mechanistic basis for the regulation of chloroplast protein import in response to environmental stress signals, with the SP1 E3 ligase being a central component of the regulatory mechanism. Furthermore, they clearly demonstrate the physiological significance of such regulation. When the data are considered in conjunction with previous results showing that protein import is regulated in response to temperature and developmental cues [5, 10, 30], a picture of chloroplast protein import as a dynamically regulated process emerges. Such regulation may help to finely tune the composition of the chloroplast proteome, ensuring that it is optimally matched to varying environmental and developmental circumstances. It would complement well-known nuclear photosynthetic gene expression responses, so that both transcriptional and post-translational regulatory steps are required for adaptation to environmental changes. That SP1 overexpression substantially reduces ROS accumulation under stress and significantly promotes stress tolerance (as measured by the greening, developmental progression, and survival) suggests potential applications in agriculture that may help to achieve important food security targets in an increasingly uncertain future [7, 25].

SUPPLEMENTAL INFORMATION

Supplemental Information includes Supplemental Experimental Procedures and four figures and can be found with this article online at http://dx.doi.org/10.1016/j.cub.2015.08.015.

ACKNOWLEDGMENTS

We thank Sean Maguire, Pedro Bota, and Ramesh Patel for technical support; Sybille Kubis for the OE33tp-CFP construct; Henrik Scheller for the PsaD antibody; and the Salk Institute Genomic Analysis Laboratory for T-DNA insertion lines. This work was supported by grants BB/H008039/1 and BB/K018442/1 from the Biotechnology and Biological Sciences Research Council (BBSRC). The role of SP1 in stress tolerance is covered by US patent application number 16/643507.

Received: February 2, 2015
Revised: July 15, 2015
Accepted: August 7, 2015
Published: September 17, 2015

REFERENCES

1. Jarvis, P., and López-Juez, E. (2013). Biogenesis and homeostasis of chloroplasts and other plastids. Nat. Rev. Mol. Cell Biol. *14*, 787–802.
2. Jensen, P.E., and Leister, D. (2014). Chloroplast evolution, structure and functions. F1000Prime Rep. *6*, 40.
3. Shi, L.X., and Theg, S.M. (2013). The chloroplast protein import system: from algae to trees. Biochim. Biophys. Acta *1833*, 314–331.
4. Paila, Y.D., Richardson, L.G., and Schnell, D.J. (2015). New insights into the mechanism of chloroplast protein import and its integration with protein quality control, organelle biogenesis and development. J. Mol. Biol. *427*, 1038–1060.
5. Ling, Q., Huang, W., Baldwin, A., and Jarvis, P. (2012). Chloroplast biogenesis is regulated by direct action of the ubiquitin-proteasome system. Science *338*, 655–659.
6. Kessler, F. (2012). Plant science. Chloroplast delivery by UPS. Science *338*, 622–623.
7. Witcombe, J.R., Hollington, P.A., Howarth, C.J., Reader, S., and Steele, K.A. (2008). Breeding for abiotic stresses for sustainable agriculture. Philos. Trans. R. Soc. Lond. B Biol. Sci. *363*, 703–716.
8. Saibo, N.J., Lourenço, T., and Oliveira, M.M. (2009). Transcription factors and regulation of photosynthetic and related metabolism under environmental stresses. Ann. Bot. (Lond.) *103*, 609–623.
9. Taylor, N.L., Tan, Y.F., Jacoby, R.P., and Millar, A.H. (2009). Abiotic environmental stress induced changes in the *Arabidopsis thaliana* chloroplast, mitochondria and peroxisome proteomes. J. Proteomics *72*, 367–378.
10. Dutta, S., Mohanty, S., and Tripathy, B.C. (2009). Role of temperature stress on chloroplast biogenesis and protein import in pea. Plant Physiol. *150*, 1050–1061.
11. Aronsson, H., Boij, P., Patel, R., Wardle, A., Töpel, M., and Jarvis, P. (2007). Toc64/OEP64 is not essential for the efficient import of proteins into chloroplasts in *Arabidopsis thaliana*. Plant J. *52*, 53–68.
12. Hruz, T., Laule, O., Szabo, G., Wessendorp, F., Bleuler, S., Oertle, L., Widmayer, P., Gruissem, W., and Zimmermann, P. (2008). Genevestigator v3: a reference expression database for the meta-analysis of transcriptomes. Adv. Bioinforma. *2008*, 420747.
13. Basnayake, B.M.V.S., Li, D., Zhang, H., Li, G., Virk, N., and Song, F. (2011). Arabidopsis DAL1 and DAL2, two RING finger proteins homologous to *Drosophila* DIAP1, are involved in regulation of programmed cell death. Plant Cell Rep. *30*, 37–48.
14. Nagata, T., Todoriki, S., Masumizu, T., Suda, I., Furuta, S., Du, Z., and Kikuchi, S. (2003). Levels of active oxygen species are controlled by ascorbic acid and anthocyanin in *Arabidopsis*. J. Agric. Food Chem. *51*, 2992–2999.
15. Richards, S.L., Laohavisit, A., Mortimer, J.C., Shabala, L., Swarbreck, S.M., Shabala, S., and Davies, J.M. (2014). Annexin 1 regulates the H_2O_2-induced calcium signature in *Arabidopsis thaliana* roots. Plant J. *77*, 136–145.
16. Qiu, Q.S., Guo, Y., Dietrich, M.A., Schumaker, K.S., and Zhu, J.K. (2002). Regulation of SOS1, a plasma membrane Na^+/H^+ exchanger in *Arabidopsis thaliana*, by SOS2 and SOS3. Proc. Natl. Acad. Sci. USA *99*, 8436–8441.
17. Zhu, J., Fu, X., Koo, Y.D., Zhu, J.K., Jenney, F.E., Jr., Adams, M.W., Zhu, Y., Shi, H., Yun, D.J., Hasegawa, P.M., and Bressan, R.A. (2007). An enhancer mutant of *Arabidopsis salt overly sensitive 3* mediates both ion homeostasis and the oxidative stress response. Mol. Cell. Biol. *27*, 5214–5224.
18. Pesaresi, P., Masiero, S., Eubel, H., Braun, H.P., Bhushan, S., Glaser, E., Salamini, F., and Leister, D. (2006). Nuclear photosynthetic gene expression is synergistically modulated by rates of protein synthesis in chloroplasts and mitochondria. Plant Cell *18*, 970–991.
19. Mittler, R. (2002). Oxidative stress, antioxidants and stress tolerance. Trends Plant Sci. *7*, 405–410.
20. Eckart, K., Eichacker, L., Sohrt, K., Schleiff, E., Heins, L., and Soll, J. (2002). A Toc75-like protein import channel is abundant in chloroplasts. EMBO Rep. *3*, 557–562.
21. Mowla, S.B., Cuypers, A., Driscoll, S.P., Kiddle, G., Thomson, J., Foyer, C.H., and Theodoulou, F.L. (2006). Yeast complementation reveals a role for an *Arabidopsis thaliana* late embryogenesis abundant (LEA)-like protein in oxidative stress tolerance. Plant J. *48*, 743–756.
22. Yu, J., Chen, S., Zhao, Q., Wang, T., Yang, C., Diaz, C., Sun, G., and Dai, S. (2011). Physiological and proteomic analysis of salinity tolerance in *Puccinellia tenuiflora*. J. Proteome Res. *10*, 3852–3870.

23. Kubis, S., Baldwin, A., Patel, R., Razzaq, A., Dupree, P., Lilley, K., Kurth, J., Leister, D., and Jarvis, P. (2003). The Arabidopsis *ppi1* mutant is specifically defective in the expression, chloroplast import, and accumulation of photosynthetic proteins. Plant Cell *15*, 1859–1871.

24. Jarvis, P., Chen, L.J., Li, H., Peto, C.A., Fankhauser, C., and Chory, J. (1998). An *Arabidopsis* mutant defective in the plastid general protein import apparatus. Science *282*, 100–103.

25. Godfray, H.C.J., Beddington, J.R., Crute, I.R., Haddad, L., Lawrence, D., Muir, J.F., Pretty, J., Robinson, S., Thomas, S.M., and Toulmin, C. (2010). Food security: the challenge of feeding 9 billion people. Science *327*, 812–818.

26. Ling, Q., and Jarvis, P. (2013). Dynamic regulation of endosymbiotic organelles by ubiquitination. Trends Cell Biol. *23*, 399–408.

27. Froehlich, J. (2011). Studying Arabidopsis envelope protein localization and topology using thermolysin and trypsin proteases. Methods Mol. Biol. *774*, 351–367.

28. Xu, T., Lee, K., Gu, L., Kim, J.I., and Kang, H. (2013). Functional characterization of a plastid-specific ribosomal protein PSRP2 in *Arabidopsis thaliana* under abiotic stress conditions. Plant Physiol. Biochem. *73*, 405–411.

29. Walley, J., Xiao, Y., Wang, J.Z., Baidoo, E.E., Keasling, J.D., Shen, Z., Briggs, S.P., and Dehesh, K. (2015). Plastid-produced interorgannellar stress signal MEcPP potentiates induction of the unfolded protein response in endoplasmic reticulum. Proc. Natl. Acad. Sci. USA *112*, 6212–6217.

30. Teng, Y.S., Chan, P.T., and Li, H.M. (2012). Differential age-dependent import regulation by signal peptides. PLoS Biol. *10*, e1001416.

Current Biology
Supplemental Information

Regulation of Chloroplast Protein Import by the Ubiquitin E3 Ligase SP1 Is Important for Stress Tolerance in Plants

Qihua Ling and Paul Jarvis

Figure S1

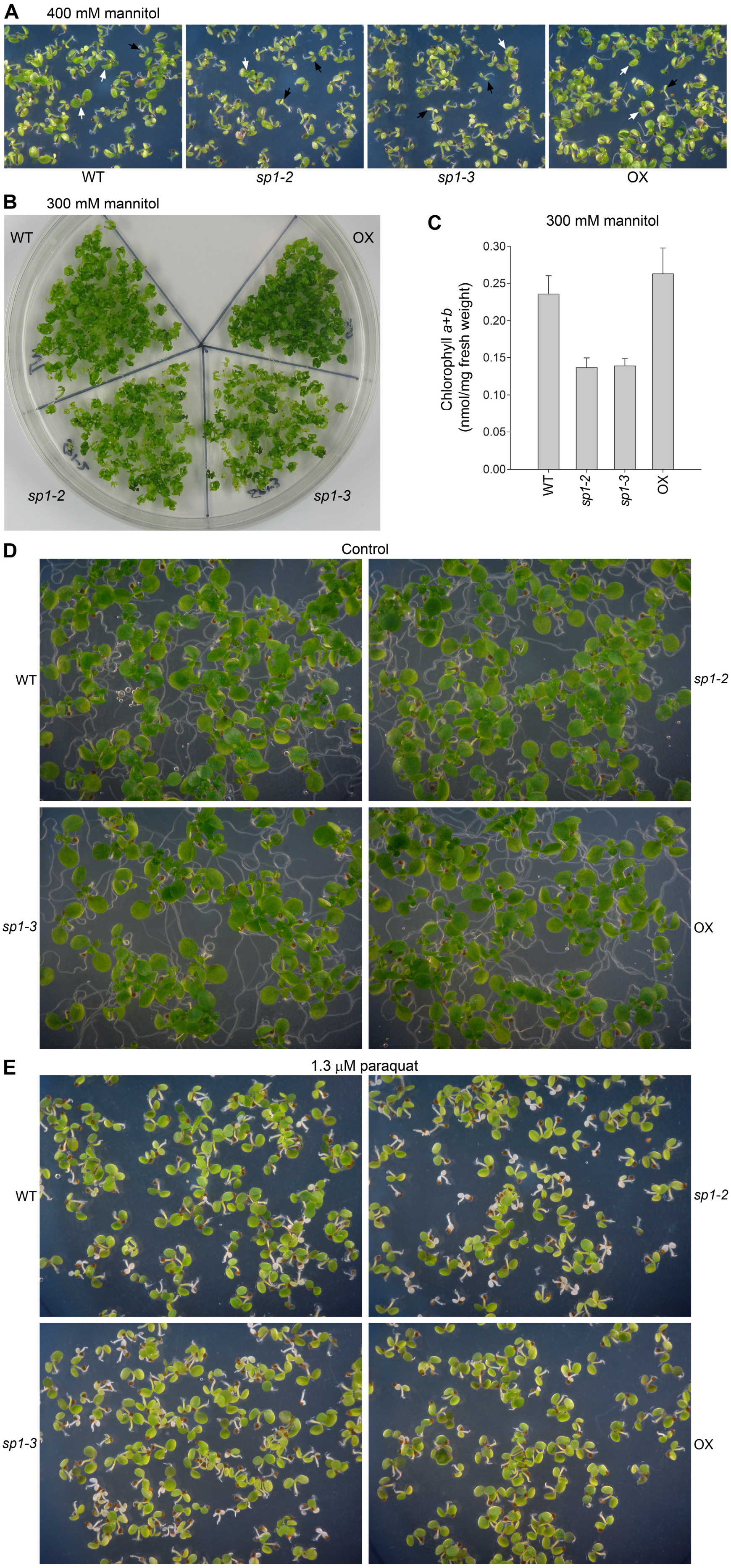
A
400 mM mannitol
WT
sp1-2
sp1-3
OX
B
300 mM mannitol
WT
OX
sp1-2
sp1-3
C
300 mM mannitol
Chlorophyll a+b (nmol/mg fresh weight)
0.30
0.25
0.20
0.15
0.10
0.05
0.00
WT
sp1-2
sp1-3
OX
D
Control
WT
sp1-2
sp1-3
OX
E
1.3 μM paraquat
WT
sp1-2
sp1-3
OX

Figure S1. SP1 Expression Levels Influence Abiotic Stress Tolerance

(A) Tolerance of strong osmotic stress. Wild type, *sp1* mutant, and SP1 overexpressor (OX) plants were germinated on medium containing 400 mM mannitol, and the plants were allowed to grow for 3 weeks prior to photography (A) and scoring for a measure of stress tolerance (i.e., development to the point of primary leaf emergence; see Figure 1D). Typical developed plants are indicated with white arrows; typical undeveloped plants are indicated with black arrows.

(B and C) Tolerance of moderate osmotic stress. The same genotypes were germinated on medium containing 300 mM mannitol, to apply a more moderate stress, and the plants were allowed to grow for 3 weeks prior to photography (B) and scoring for an alternative measure of stress tolerance (C). In this case, stress tolerance was estimated by quantifying the amount of chlorophyll per unit fresh weight in the plants, which was not possible at higher mannitol concentrations due to the failure of many plants to develop. Values shown are means (n=3), and error bars indicate SEM.

(D and E) Appearance of *sp1* mutant and sp1 overexpressor plants under normal and oxidative stress conditions. The indicated genotypes were germinated on standard MS medium (D) or on medium containing 1.3 µM paraquat (E), and the plants were allowed to grow for 10 days prior to photography. Under the normal (non-stressful) growth conditions applied in D, neither the mutant nor the overexpressor plants displayed any phenotypic differences from wild type. Under the oxidative stress conditions applied in E, a variable proportion of the plants died (dead plants appear small and white) dependent upon the level of SP1 activity. Images shown in Figure 1E correspond to small regions of the images shown in E here, which were selected as being representative of the trends revealed in Figure 1F.

Figure S2

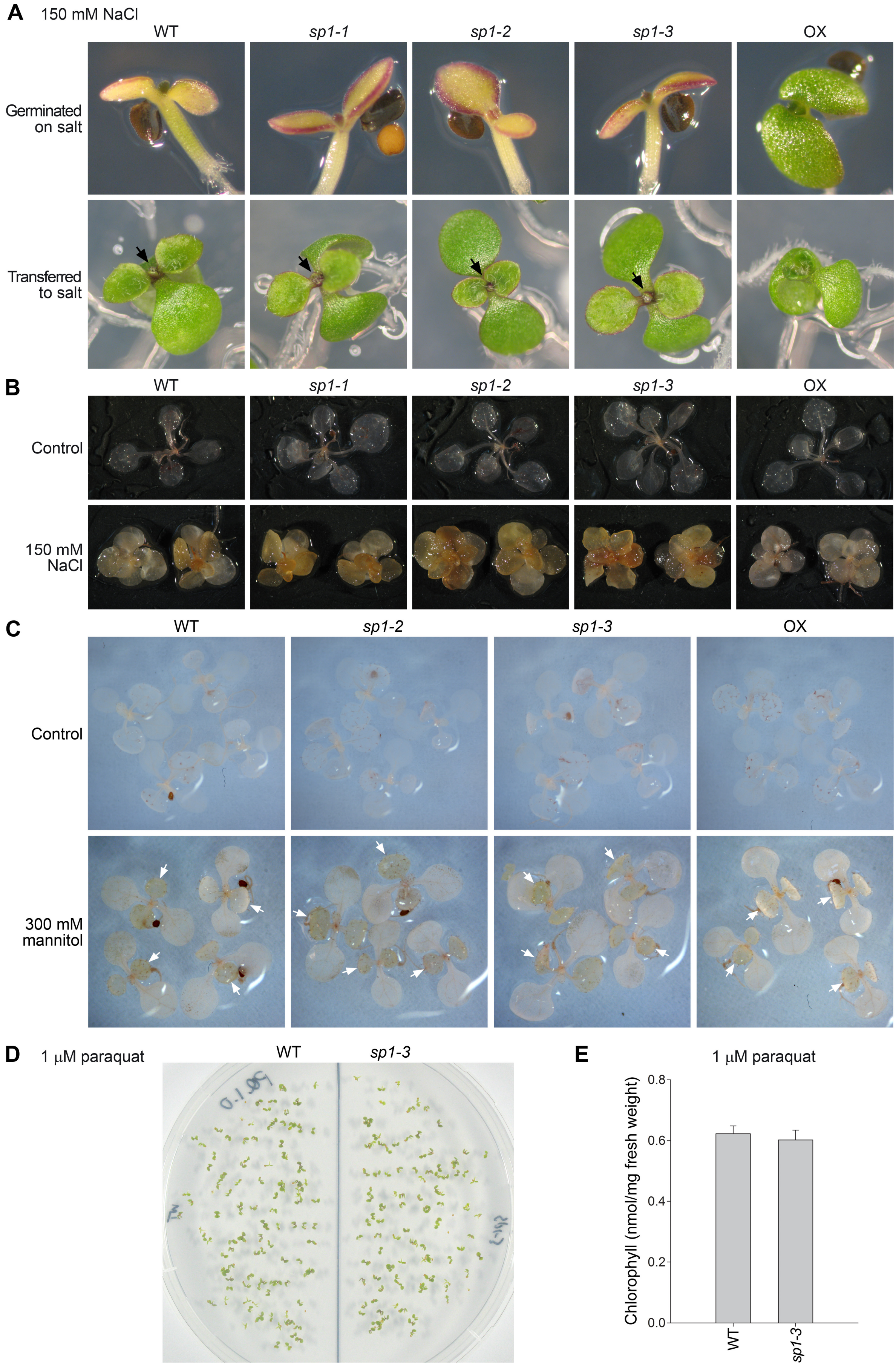

A
150 mM NaCl
WT
sp1-1
sp1-2
sp1-3
OX
Germinated on salt
Transferred to salt
B
WT
sp1-1
sp1-2
sp1-3
OX
Control
150 mM NaCl
C
WT
sp1-2
sp1-3
OX
Control
300 mM mannitol
D
1 μM paraquat
WT
sp1-3
E
1 μM paraquat
Chlorophyll (nmol/mg fresh weight)
0.0
0.2
0.4
0.6
0.8
WT
sp1-3

Figure S2. Effects of SP1 on the Tolerance of Salt and Osmotic Stresses are Linked to ROS

(A) Anthocyanin accumulation following salt stress. The indicated genotypes were germinated on medium containing 150 mM NaCl, or on standard medium and allowed to grow for 4 days before transferral to 150 mM NaCl. Respectively, the plants were allowed to grow for 14 days (upper panels), or for 10 further days following transfer (lower panels). In both cases, accumulation of the purple, stress-related pigment anthocyanin (an antioxidant) was clearly apparent in the leaves of wild type and *sp1* mutant plants, but not in SP1 overexpressor (OX) plants; in the transferred plants, such pigmentation was particularly prominent in the newly emerging true leaves (see arrows). Following germination on saline medium, wild-type and *sp1* mutant plants typically failed to green and were stunted, whereas OX plants were remarkably healthy. Moreover, we consistently observed that SP1 overexpressor plants experienced a developmental delay immediately following transfer to salt stress conditions (hence the smaller size of the OX plant shown). This observation is consistent with our hypothesis that SP1 acts to attenuate photosynthetic activity under stress conditions, which may initially retard growth but ultimately proves beneficial if the stress persists.

(B) Staining for hydrogen peroxide accumulation following salt stress. The same genotypes were stressed exactly as described in A (lower panels), by transferral to medium containing 150 mM NaCl (or to medium lacking NaCl as a control), except that in this case the transferred plants were allowed to grow on for 3 weeks prior to analysis. Stressed and control, mock-treated plants were stained with 3,3'-diaminobenzidine (DAB), and representative images are shown. Brown DAB staining is indicative of H_2O_2 accumulation.

(C) Staining for hydrogen peroxide accumulation following osmotic stress. The indicated genotypes were germinated on standard medium and allowed to grow for 8 days before transferral to medium containing 300 mM mannitol (or medium lacking mannitol as a control). The transferred plants were kept under standard conditions for a further 3 days prior to analysis. Stressed and control, mock-treated plants were stained with DAB, and representative images are shown. Under these conditions, staining was most apparent in the true leaves (see arrows).

(D and E) phenotype of *sp1* single mutant plants under moderate oxidative stress. Wild-type and *sp1* single mutant plants were germinated on medium containing 1 µM paraquat and allowed to grow for 7 days (i.e., identical conditions to those employed in Figures 2C and 2D). At the end of the treatment period, the plants were photographed (D) or subjected to chlorophyll content analysis (E). Values shown in E are means (n=3), and error bars indicate SEM. Phenotypic similarity of the *sp1* mutant to wild type under these conditions indicates that the enhanced ROS sensitivity seen in the *sp1 sos2* and *sp1 enh1* double mutants (Figures 2C and 2D) was synergistic and not an additive effect, and this implies involvement of SP1 in ROS regulation.

Figure S3

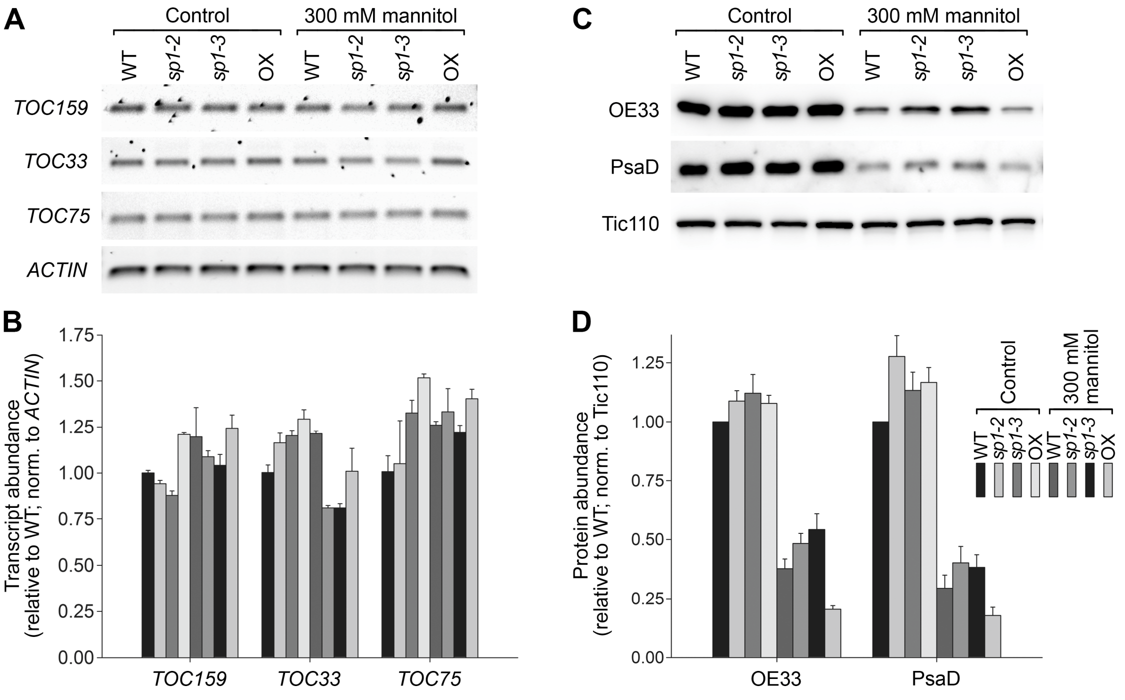

Figure S3. Analyses of the Levels of TOC Transcripts and of Photosynthetic Apparatus Proteins Under Stress Conditions.

(A and B) TOC component mRNA levels do not change in response to osmotic stress. The indicated genotypes were germinated on standard medium and allowed to grow for 7 days before transferral to medium containing 300 mM mannitol (or medium lacking mannitol as a control). The transferred plants were kept under standard conditions for a further 2 days, to deliver short-term osmotic stress as in Figure 3A, prior to analysis. Total RNA was extracted from each of the samples, and subjected to both semi-quantitative (A) and quantitative (B) RT-PCR analysis. Equivalent actin data were employed for normalization purposes. Shading of the bars in B is as defined in panel D.

(C and D). SP1-dependent depletion of photosynthetic proteins under long-term osmotic stress. The indicated genotypes were germinated on standard medium and allowed to grow for 4 days before transferral to medium containing 300 mM mannitol (or medium lacking mannitol as a control). The transferred plants were kept under standard conditions for a further 10 days prior to analysis. This longer-term osmotic stress treatment was designed to enable detection of secondary effects of the stress. Total protein extracts were prepared from the plant material, and equal amounts from each genotype were analysed by immunoblotting using antibodies raised against a core component of each photosystem (PSII: OE33 [PsbO]; PSI: PsaD) (C). Specific bands detected by immunoblotting in C (and in five additional, similar experiments) were quantified, and the values obtained were normalized relative to corresponding data for Tic110; data for each genotype were normalized relative to the wild type (D). Tic110 levels were found to be remarkably stable across the genotypes and conditions, in this study (Figure 3) and in previous studies [S1], and so normalizing relative to Tic110 was justified. Values shown are means (n=6), and error bars indicate SEM.

Figure S4

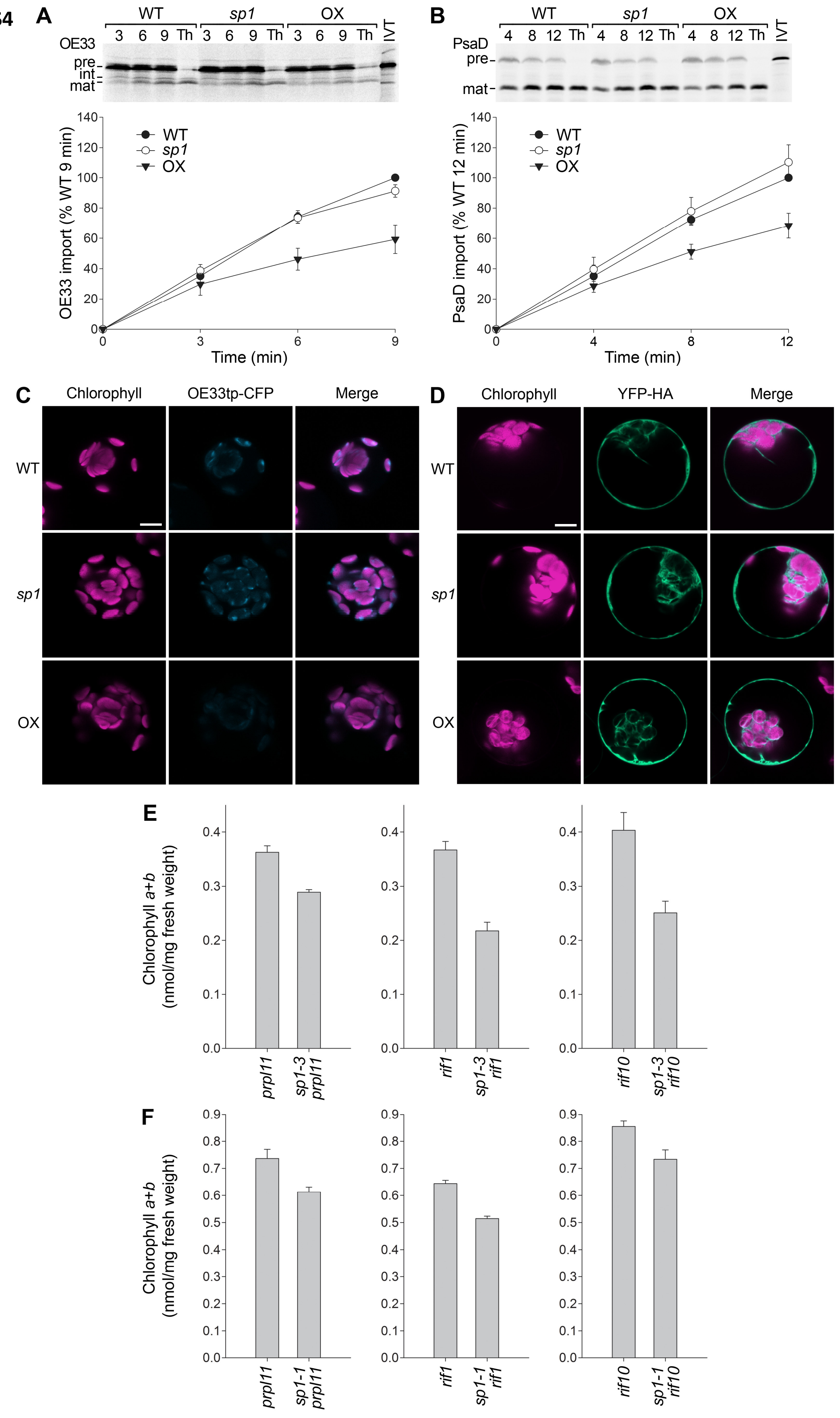
A
WT
sp1
OX
3 6 9 Th 3 6 9 Th 3 6 9 Th
IVT
OE33
pre
int
mat
OE33 import (% WT 9 min)
Time (min)
B
PsaD
4 8 12 Th 4 8 12 Th 4 8 12 Th
PsaD import (% WT 12 min)
C
Chlorophyll
OE33tp-CFP
Merge
D
YFP-HA
E
Chlorophyll a+b (nmol/mg fresh weight)
prpl11
sp1-3 prpl11
rif1
sp1-3 rif1
rif10
sp1-3 rif10
F
sp1-1 prpl11
sp1-1 rif1
sp1-1 rif10

Figure S4. Effects of Altered SP1 Levels on Chloroplast Protein Import and Genetic Interactions between *sp1* and Mutations Affecting the Plastid Genetic System

(A and B) Analysis of protein import in vitro under normal conditions. Chloroplasts isolated from unstressed 10-day-old wild-type, *sp1-3* and SP1 overexpressor (OX) seedlings (normalized according to chlorophyll amount) were incubated with in vitro translated, radiolabelled precursors of OE33 (A) and PsaD (B) under import conditions. Import was allowed to proceed for the time periods shown, and then the precursor (p), intermediate (i) and mature (m) protein forms associated with the chloroplasts were analysed by SDS-PAGE and phosphorimaging. Representative images are shown. The final time-point in each case was conducted in duplicate, with one sample being treated with thermolysin (Th) protease to remove un-imported precursor. The last lane shows an aliquot of the in vitro translation (IVT) product used in each case. Bands corresponding to the intermediate and mature forms of OE33, and to the mature form of PsaD, were quantified, and the values were normalized relative to those obtained for wild-type chloroplasts at the final time-point. Values shown are means (n=3-6), and error bars indicate SEM.

(C and D) Confirmation of the subcellular localization of fluorescent proteins following transfection for the in vivo import analysis. Protoplasts isolated from wild-type, *sp1-3* and SP1 overexpressor (OX) rosette leaves were transfected with plasmids encoding the following chimeric proteins: transit peptide of OE33 fused to CFP (OE33tp-CFP; C), and YFP fused to the haemagglutinin tag (YFP-HA; D). Transfected protoplasts were incubated for ~15 hours prior to analysis by confocal microscopy. Chlorophyll autofluorescence was employed to determine the localization of the CFP and YFP fluorescent signals relative to chloroplasts: CFP was clearly chloroplast localized, whereas YFP was cytosolic. The intensity of CFP signals was dependent upon the protoplast genotype (highest in *sp1* mutant; lowest in SP1 overexpressor, OX), whereas YFP signal intensity was independent of genotype. Scale bars represent 10 µm.

(E and F) Genetic interactions between *sp1* and mutations affecting the plastid genetic system. The *sp1-1* and *sp1-3* mutants were crossed to three different mutants with defects in aspects of plastid gene expression: *prpl11*, *rif1* and *rif10* [S2, S3, S4]. Double homozygous mutants were identified, and their phenotypes were carefully compared with those of the corresponding single mutants after 7 (E) and 10 (F) days of growth, by making chlorophyll measurements. The chlorotic phenotype of each plastid gene expression mutant was significantly enhanced by the *sp1* mutation (which in isolation does not cause any visible abnormal phenotypes under normal growth conditions). It has been shown that the inhibition of plastid gene expression can trigger retrograde signalling to suppress the expression of photosynthetic genes, which is similar to the consequence of stress treatment [S5, S6]. However, this down-regulation is not very efficient when the defect is not strong, as illustrated by the *prpl11* single mutant [S7]. Thus, in the context of the plastid gene expression defects of the *prpl11*, *rif1* and *rif10* mutants, SP1 might help to efficiently down-regulate photosynthetic proteins by controlling protein import, acting in a way that is complementary to retrograde signalling. Defective plastid gene expression may compromise carbon fixation, e.g. due to insufficiency of Rubisco large subunit [S2], and this could lead to excessive ROS production. Our results show that failure to properly regulate protein import in these mutant backgrounds has adverse consequences for plants, possibly due to the accumulation of ROS. This indicates that protein import is finely regulated to cope with different states of chloroplast biogenesis.

Supplemental Experimental Procedures

Plant Growth Conditions and Physiological Studies

All *Arabidopsis thaliana* plants used in this work were of the Columbia-0 (Col-0) ecotype. The *sp1-1*, *sp1-2*, *sp1-3*, SP1 overexpressor (OX), *ppi1-1*, *enh1-2*, *sos2-2* and *sos3-1* mutant and transgenic lines have all been reported previously [S1, S8, S9, S10]. For in vitro growth, seeds were surface sterilized, sown on Murashige-Skoog (MS) agar medium in petri plates, cold-treated at 4°C, and thereafter kept in a growth chamber, as described previously [S11]. All plants were grown under a long-day cycle (16 hours light, 8 hours dark), except those used for protoplast isolation which were grown under short day conditions (8 hours light, 16 hours dark). Chlorophyll measurements were performed following *N*,*N'*-dimethylformamide (DMF) extraction using a spectrophotometer [S12, S13, S14].

For the stress experiments, all seeds of the different genotypes were harvested at the same time. Plants were either germinated directly on, or transferred from normal MS agar medium to, MS agar medium supplemented with 150 mM NaCl, 300-400 mM mannitol, or 1.0-1.3 µM paraquat (added before pouring the plates) for salt, osmotic or oxidative stress treatment, respectively. In general, germinating directly on stress medium was used to assess the stress phenotype, while transfer to stress medium was used to study the mechanism of stress tolerance (to avoid possible non-specific effects caused by phenotypical differences between genotypes). Sucrose was omitted from the MS medium in all stress experiments, except for those involving direct germination on NaCl plates (which contained 0.5% sucrose), to minimize possible non-specific effects [S15]. For assessing growth performance or survival rate, at least three experiments were performed, and ~50-300 seedlings per genotype were analysed in each experiment.

For Figures 1A-1C, plants were germinated on medium containing 150 mM NaCl and allowed to grow under standard conditions for 14 days prior to photography and scoring for a measure of stress tolerance and germination efficiency; stress tolerance was estimated by counting the number of green individuals per genotype, and expressing these numbers as a percentage of the total number of germinated plants per genotype. For Figure 1D, plants were germinated on medium containing 400 mM mannitol and allowed to grow for 3 weeks prior to scoring for a measure of stress tolerance; stress tolerance was estimated by counting the number of plants that had developed to the point of primary leaf emergence, and expressing these numbers as a percentage of the total number of plants per genotype (germination efficiency was essentially 100% in all genotypes; data not shown). For Figures 1E and 1F, plants were germinated on medium containing 1.3 µM paraquat and allowed to grow for 10 days prior to photography and scoring for death or survival as a measure

of stress sensitivity; dead plants were very small and white, whereas surviving plants had expanded, green cotyledons (germination efficiency was essentially 100% in all genotypes; data not shown).

For Figures 3A and 3B, plants were germinated on standard medium and allowed to grow for 7 days before transferral to medium containing 300 mM mannitol (or medium lacking mannitol as a control). The transferred plants were kept under standard conditions for a further 2 days prior to analysis. This short-term osmotic stress treatment was designed to enable detection of the primary effects of the stress upon analysis by immunoblotting.

Generation and Analysis of Double Mutants

Double homozygous mutants combining *sp1-3* with *enh1-2*, *sos2-2* or *sos3-1* were identified in the F2 or F3 generation of the corresponding crosses by PCR testing. The *sp1-3* mutation was identified by gene-specific primers 5’-GCTGTCAAGGACGATATTG-3’ and 5’-CAGACAAGACTCAAGTAGAGAATG-3’, and the former in combination with the left border T-DNA-specific primer 5’-GCGTGGACCGCTTGCTGCAACT-3’. The *enh1-2* and *sos3-1* mutations were identified as described previously [S9, S10]. The *sos2-2* mutation was identified by dCAPS analysis involving PCR with primers 5’-TTGGATGATATTCGTGCAGATC-3’ and 5’-TTAACATTTAAATGGAATTGACC-3’ followed by digestion with restriction enzyme *Bgl*II, and verified by DNA sequencing. The corresponding single homozygous mutants were also identified, as siblings in the same generations, and these were employed as controls for the phenotypical comparisons. For Figures 2C and 2D, plants were subjected to moderate oxidative stress by germination and growth for 7 days on medium containing 1 µM paraquat; at the end of the treatment period, the plants were photographed or subjected to chlorophyll content analysis. Enhanced phenotype severity in the *sp1 sos2* and *sp1 enh1* double mutants, but not in *sp1 sos3*, revealed synergistic genetic interactions and implied involvement of SP1 in ROS regulation like SOS2 and ENH1, but via a different mechanism.

ROS Detection

The detection of hydrogen peroxide was performed by staining with 3,3’-diaminobenzidine (DAB) (Sigma-Aldrich) using a modified published method [S16]. Briefly, seedlings growing on normal MS medium for 3 (for NaCl stress) or 8 (for mannitol and paraquat stresses) days (under these conditions, seedlings of all the genotypes appeared normal and developed similarly) were transferred to corresponding stress plates for 3 weeks (for NaCl stress), 3 days (for mannitol stress), or 2 days (for paraquat stress). Then, the shoots of the seedlings were collected and infiltrated for 10

minutes using a gentle vacuum with freshly-made staining solution (1 mg/mL DAB, 0.05% (v/v) Tween 20, and 10 mM sodium phosphate buffer, pH 7.0). For each experiment, 5-10 seedlings were used and the experiment was repeated at least twice. Infiltrated samples were incubated in the dark at room temperature with gentle shaking for 6 hours to overnight. The staining reaction was then terminated by replacing the staining solution with bleaching solution (ethanol:glycerol:acetic acid, 3:1:1 by volume) and incubation in a water bath at 95°C for 15 min. Then, the samples were incubated with fresh bleaching solution at room temperature until the chlorophyll was completely depleted, and transferred to ethanol:glycerol (4:1 by volume) for photography.

For Figures 2A and 2B, 8-day-old plants germinated on standard medium were transferred to medium containing 1 μM paraquat (or medium lacking paraquat as a control) and analysed after a further 2 days; such treatment generated moderate levels of oxidative stress without presenting survival issues, did not cause visibly different stress phenotypes in the different genotypes, and was found to be optimal for this analysis. For quantification, ImageJ was used to establish a threshold of DAB staining over the background, and to calculate the area of DAB staining versus the total area of the shoot.

Semi-Quantitative and Quantitative RT-PCR

RNA extraction, reverse transcription, and semi-quantitative or quantitative RT-PCR were performed as described previously [S17]. The primers used for PCR amplification were: *ACTIN*, 5'-CAAGCAGCATGAAGATTAAGGTCGTT-3' and 5'-CTTGGAGATCCACATCTGCTGGAAT-3'; *TOC33*, 5'-AATGGTGAAGCGTGGATC-3' and 5'-TGCTCCTTGAATCATCTTAACG-3'; *TOC159*, 5'-AACTCTTGAAGTGGCTAATAAGT-3' and 5'-ACAACCTCTGGCTCTACA-3'; and *TOC75*, 5'-TCGCATCTCCACTCAATC-3' and 5'-GTCTCTGTATCTCGGTTAGG-3'; and *CFP*, 5'-TACAACTACAACAGCCACAA-3' and 5'-CGGATCTTGAAGTTCACCTT-3'. Gene expression data were normalized using the data for *ACTIN*. To exclude the possibility of DNA contamination (genomic or transfected plasmid) in the RNA samples, which was especially important for the analysis presented in Figure 4F, appropriate amounts of RNA (equivalent to those used for cDNA synthesis) were similarly tested in control PCR experiments. No significant amplification signal was detected, eliminating the possibility of DNA contamination that might affect quantification.

Plasmid Construction and Protoplast Transient Assays

For the construct of OE33tp-CFP, the oxygen evolving complex 33 kD subunit (OE33; At5g66570) transit peptide was amplified by PCR from Col-0 cDNA, and then cloned into p2GWC7 [S18] using Gateway technology to produce a C-terminal fusion to CFP. OE33 has a bipartite targeting signal comprising a transit peptide of 29 amino acids followed by a thylakoid lumen targeting signal of 56 residues. Sequence encoding the whole transit peptide and part of the lumen targeting domain (37 amino acids) was amplified, to enable targeting of CFP protein into the chloroplast stroma in protoplasts. The YFP-HA construct has been described previously [S1].

Protoplast isolation and transient assays were carried out as described previously [S19], except that a culture buffer (500 mM mannitol, 4 mM 4-morpholineethanesulfonic acid [MES], pH 5.6, and 10 mM KCl) was used to replace W5 buffer for the final overnight incubation, to facilitate the following protein extraction. Protoplasts were isolated from plants grown under short day condition for 7-8 weeks. For either XFP fluorescence or immunoblotting assays, 0.1 mL (10^5) of protoplasts were transfected with 5 µg DNA. After incubation in the dark after 15 hours, the protoplasts were analysed, either under a microscope for XFP fluorescence or by immunoblotting. For immunoblotting assays, protoplasts were pelleted at 100g for 2 minutes and the pellet was added directly to 2× SDS-PAGE loading buffer (50 mM Tris-HCl, pH 6.8, 20% glycerol, 1% sodium dodecyl sulphate [SDS], and 0.1 M dithiothreitol), followed by SDS-PAGE and immunoblotting.

For Figures 4C-4H, protoplasts isolated from rosette leaves of wild-type, *sp1-3* and SP1 overexpressor plants were transfected with plasmids encoding either one or the other of the following chimeric proteins: transit peptide of OE33 fused to CFP (OE33tp-CFP), and YFP fused to the haemagglutinin tag (YFP-HA) as a control. Transfected protoplasts were analysed by immunoblotting to detect the transiently expressed protein as well as native Toc75 or Tic110. Specific bands detected in repeated experiments were quantified, and the values for each protein were normalized relative to corresponding Tic110 values, prior to further normalization relative to the wild type. For Figure 4F, RNA was extracted from OE33tp-CFP-transfected protoplasts (three independent samples per genotype), and subjected to quantitative RT-PCR analysis using *CFP*- and *ACTIN*-specific primers. The *CFP* data were normalized relative to corresponding *ACTIN* data, prior to further normalization relative to the wild type.

In Vitro Translation, Chloroplast Isolation, and Import

Previously described clones carrying the full-length cDNAs of OE33 (119E10T7) or PsaD (307C9T7) were employed to amplify template using M13 primers [S20]. The in vitro transcription/translation procedure was described previously [S13, S21]. For normal conditions, chloroplasts were isolated from 10-day-old plants grown in vitro on standard MS agar medium. For stress conditions, chloroplasts were isolated from plants that had been grown on standard MS agar medium for 8 days and then transferred into liquid MS medium supplemented with 200 mM mannitol for 2 days (under these conditions, we found the yield of chloroplasts and the degree of stress to be optimal for import assays; more severe treatments adversely affected yield and/or import competence of the organelles). Isolations and protein import were performed as described previously [S11, S22]. Thermolysin treatments were conducted using standard procedures [S1, S23]. Band intensities were quantified using Aida software (Raytest).

For Figures 4A and 4B, chloroplasts isolated from osmotically-stressed 10-day-old wild-type, *sp1-3* and SP1 overexpressor seedlings (normalized according to chlorophyll amount) were incubated with in vitro translated, radiolabelled precursors of OE33 and PsaD under import conditions. The final time-point in each case was conducted in duplicate, with one sample being treated with thermolysin protease to remove un-imported precursor. Bands corresponding to the intermediate and mature forms of OE33, and to the mature form of PsaD, were quantified, and the values were normalized relative to those obtained for wild-type chloroplasts at the final time-point.

Immunoblotting

Immunoblotting was performed as described previously [S24, S25] with minor modifications. Primary antibodies were anti-Toc75-III [S17], anti-atToc33 peptide antibody [S26], anti-atTic40 [S17], anti-atTic110 [S27], anti-atToc159 [S28], anti-OEP80 [S29], anti-OE33 [S1], anti-PsaD [S30], anti-HA (Sigma), and anti-YFP (BD Biosciences). Secondary antibodies were anti-rabbit IgG conjugated with either alkaline phosphatase (Sigma) or horseradish peroxidase (Santa Cruz Biotechnology). Chemiluminescence was detected using ECL Plus Western Blotting Detection Reagent (GE Healthcare) and an LAS-4000 imager (Fujifilm, GE Healthcare). Band intensities were quantified using Aida software (Raytest).

For Figures 3A and 3B, specific bands detected by immunoblotting in four similar experiments were quantified, and the values obtained were normalized relative to corresponding data for the

Coomassie-stained Rubisco large subunit band; data for each genotype were normalized relative to the wild type.

Confocal Laser-Scanning Microscopy

For the imaging of CFP, YFP and chlorophyll fluorescence signals, protoplasts were examined using a Zeiss LSM 510 META laser-scanning microscope (Carl Zeiss Ltd., Welwyn Garden City, Herts, UK). The CFP fluorescence was excited with a 458-nm excitation line of a 25 mW argon ion laser and an HFT 458/514 primary dichroic mirror, and was detected by a 475- to 525-nm band-pass filter in the single-track facility of the microscope. To detect YFP, a 514-nm excitation from a 5 mW argon ion laser with an HFT 458/514 primary dichroic mirror and a 535- to 590-nm emission filter was used. In each case, to simultaneously detect chlorophyll autofluorescence, a NFT 635 vis long-pass filter was used. Images were processed using Zeiss LSM Image Browser. All experiments were conducted at least twice with the same results, and typical images are shown.

Underlying research materials are available from the corresponding author upon request.

Supplemental References

S1. Ling, Q., Huang, W., Baldwin, A., and Jarvis, P. (2012). Chloroplast biogenesis is regulated by direct action of the ubiquitin-proteasome system. Science *338*, 655-659.

S2. Pesaresi, P., Varotto, C., Meurer, J., Jahns, P., Salamini, F., and Leister, D. (2001). Knock-out of the plastid ribosomal protein L11 in *Arabidopsis*: effects on mRNA translation and photosynthesis. Plant J. *27*, 179-189.

S3. Flores-Perez, U., Sauret-Gueto, S., Gas, E., Jarvis, P., and Rodriguez-Concepcion, M. (2008). A mutant impaired in the production of plastome-encoded proteins uncovers a mechanism for the homeostasis of isoprenoid biosynthetic enzymes in Arabidopsis plastids. Plant Cell *20*, 1303-1315.

S4. Sauret-Güeto, S., Botella-Pavía, P., Flores-Pérez, U., Martínez-García, J.F., San Román, C., León, P., Boronat, A., and Rodríguez-Concepción, M. (2006). Plastid cues posttranscriptionally regulate the accumulation of key enzymes of the methylerythritol phosphate pathway in Arabidopsis. Plant Physiol. *141*, 75-84.

S5. Koussevitzky, S., Nott, A., Mockler, T.C., Hong, F., Sachetto-Martins, G., Surpin, M., Lim, J., Mittler, R., and Chory, J. (2007). Signals from chloroplasts converge to regulate nuclear gene expression. Science *316*, 715-719.

S6. Sun, X., Feng, P., Xu, X., Guo, H., Ma, J., Chi, W., Lin, R., Lu, C., and Zhang, L. (2011). A chloroplast envelope-bound PHD transcription factor mediates chloroplast signals to the nucleus. Nat. Commun. *2*, 477.

S7. Pesaresi, P., Masiero, S., Eubel, H., Braun, H.P., Bhushan, S., Glaser, E., Salamini, F., and Leister, D. (2006). Nuclear photosynthetic gene expression is synergistically modulated by rates of protein synthesis in chloroplasts and mitochondria. Plant Cell *18*, 970-991.

S8. Liu, J., Ishitani, M., Halfter, U., Kim, C.S., and Zhu, J.K. (2000). The *Arabidopsis thaliana SOS2* gene encodes a protein kinase that is required for salt tolerance. Proc. Natl. Acad. Sci. USA *97*, 3730-3734.

S9. Halfter, U., Ishitani, M., and Zhu, J.K. (2000). The *Arabidopsis* SOS2 protein kinase physically interacts with and is activated by the calcium-binding protein SOS3. Proc. Natl. Acad. Sci. USA *97*, 3735-3740.

S10. Zhu, J., Fu, X., Koo, Y.D., Zhu, J.K., Jenney, F.E., Jr., Adams, M.W., Zhu, Y., Shi, H., Yun, D.J., Hasegawa, P.M., et al. (2007). An enhancer mutant of *Arabidopsis salt overly sensitive 3* mediates both ion homeostasis and the oxidative stress response. Mol. Cell. Biol. *27*, 5214-5224.

S11. Aronsson, H., and Jarvis, P. (2002). A simple method for isolating import-competent *Arabidopsis* chloroplasts. FEBS Lett. *529*, 215-220.

S12. Jarvis, P., Chen, L.J., Li, H., Peto, C.A., Fankhauser, C., and Chory, J. (1998). An *Arabidopsis* mutant defective in the plastid general protein import apparatus. Science *282*, 100-103.

S13. Huang, W., Ling, Q., Bédard, J., Lilley, K., and Jarvis, P. (2011). In vivo analyses of the roles of essential Omp85-related proteins in the chloroplast outer envelope membrane. Plant Physiol. *157*, 147-159.

S14. Constan, D., Patel, R., Keegstra, K., and Jarvis, P. (2004). An outer envelope membrane component of the plastid protein import apparatus plays an essential role in *Arabidopsis*. Plant J. *38*, 93-106.

S15. Verslues, P.E., Agarwal, M., Katiyar-Agarwal, S., Zhu, J., and Zhu, J.K. (2006). Methods and concepts in quantifying resistance to drought, salt and freezing, abiotic stresses that affect plant water status. Plant J. *45*, 523-539.

S16. Daudi, A., Cheng, Z., O'Brien, J.A., Mammarella, N., Khan, S., Ausubel, F.M., and Bolwell, G.P. (2012). The apoplastic oxidative burst peroxidase in *Arabidopsis* is a major component of pattern-triggered immunity. Plant Cell *24*, 275-287.

S17. Kasmati, A.R., Töpel, M., Patel, R., Murtaza, G., and Jarvis, P. (2011). Molecular and genetic analyses of Tic20 homologues in *Arabidopsis thaliana* chloroplasts. Plant J. *66*, 877-889.

S18. Karimi, M., De Meyer, B., and Hilson, P. (2005). Modular cloning in plant cells. Trends Plant Sci. *10*, 103-105.

S19. Wu, F.H., Shen, S.C., Lee, L.Y., Lee, S.H., Chan, M.T., and Lin, C.S. (2009). Tape-Arabidopsis Sandwich - a simpler Arabidopsis protoplast isolation method. Plant Methods *5*, 16.

S20. Kubis, S., Baldwin, A., Patel, R., Razzaq, A., Dupree, P., Lilley, K., Kurth, J., Leister, D., and Jarvis, P. (2003). The Arabidopsis *ppi1* mutant is specifically defective in the expression, chloroplast import, and accumulation of photosynthetic proteins. Plant Cell *15*, 1859-1871.

S21. Baldwin, A., Wardle, A., Patel, R., Dudley, P., Park, S.K., Twell, D., Inoue, K., and Jarvis, P. (2005). A molecular-genetic study of the Arabidopsis Toc75 gene family. Plant Physiol. *138*, 715-733.

S22. Aronsson, H., and Jarvis, R.P. (2011). Rapid isolation of Arabidopsis chloroplasts and their use for in vitro protein import assays. Methods Mol. Biol. *774*, 281-305.

S23. Froehlich, J. (2011). Studying Arabidopsis envelope protein localization and topology using thermolysin and trypsin proteases. Methods Mol. Biol. *774*, 351-367.

S24. Kovacheva, S., Bédard, J., Patel, R., Dudley, P., Twell, D., Ríos, G., Koncz, C., and Jarvis, P. (2005). *In vivo* studies on the roles of Tic110, Tic40 and Hsp93 during chloroplast protein import. Plant J. *41*, 412-428.

S25. Kovacheva, S., Bédard, J., Wardle, A., Patel, R., and Jarvis, P. (2007). Further *in vivo* studies on the role of the molecular chaperone, Hsp93, in plastid protein import. Plant J. *50*, 364-379.

S26. Aronsson, H., Combe, J., and Jarvis, P. (2003). Unusual nucleotide-binding properties of the chloroplast protein import receptor, atToc33. FEBS Lett. *544*, 79-85.

S27. Aronsson, H., Combe, J., Patel, R., Agne, B., Martin, M., Kessler, F., and Jarvis, P. (2010). Nucleotide binding and dimerization at the chloroplast pre-protein import receptor, atToc33, are not essential *in vivo* but do increase import efficiency. Plant J. *63*, 297-311.

S28. Bauer, J., Chen, K., Hiltbrunner, A., Wehrli, E., Eugster, M., Schnell, D., and Kessler, F. (2000). The major protein import receptor of plastids is essential for chloroplast biogenesis. Nature *403*, 203-207.

S29. Hsu, S.C., Nafati, M., and Inoue, K. (2012). OEP80, an essential protein paralogous to the chloroplast protein translocation channel Toc75, exists as a 70-kD protein in the *Arabidopsis thaliana* chloroplast outer envelope. Plant Mol. Biol. *78*, 147-158.

S30. Andersen, B., Koch, B., and Scheller, H.V. (1992). Structural and functional analysis of the reducing side of photosystem I. Physiol. Plant. *84*, 154-161.
